# Delineating CD4 dependency of HIV-1: Adaptation to infect low level CD4 expressing target cells widens cellular tropism but severely impacts on envelope functionality

**DOI:** 10.1371/journal.ppat.1006255

**Published:** 2017-03-06

**Authors:** David Beauparlant, Peter Rusert, Carsten Magnus, Claus Kadelka, Jacqueline Weber, Therese Uhr, Osvaldo Zagordi, Corinna Oberle, Maria J. Duenas-Decamp, Paul R. Clapham, Karin J. Metzner, Huldrych F. Günthard, Alexandra Trkola

**Affiliations:** 1 Institute of Medical Virology, University of Zurich, Zurich, Switzerland; 2 Division of Infectious Diseases and Hospital Epidemiology, University Hospital Zurich, Zurich, Switzerland; 3 Program in Molecular Medicine, Biotech II, University of Massachusetts Medical School, Worcester, Massachusetts, United States of America; University of Wisconsin, UNITED STATES

## Abstract

A hallmark of HIV-1 infection is the continuously declining number of the virus’ predominant target cells, activated CD4^+^ T cells. With diminishing CD4^+^ T cell levels, the capacity to utilize alternate cell types and receptors, including cells that express low CD4 receptor levels such as macrophages, thus becomes crucial. To explore evolutionary paths that allow HIV-1 to acquire a wider host cell range by infecting cells with lower CD4 levels, we dissected the evolution of the envelope-CD4 interaction under *in vitro* culture conditions that mimicked the decline of CD4^high^ target cells, using a prototypic subtype B, R5-tropic strain. Adaptation to CD4^low^ targets proved to severely alter envelope functions including trimer opening as indicated by a higher affinity to CD4 and loss in shielding against neutralizing antibodies. We observed a strikingly decreased infectivity on CD4^high^ target cells, but sustained infectivity on CD4^low^ targets, including macrophages. Intriguingly, the adaptation to CD4^low^ targets altered the kinetic of the entry process, leading to rapid CD4 engagement and an extended transition time between CD4 and CCR5 binding during entry. This phenotype was also observed for certain central nervous system (CNS) derived macrophage-tropic viruses, highlighting that the functional perturbation we defined upon *in vitro* adaptation to CD4^low^ targets occurs *in vivo*. Collectively, our findings suggest that CD4^low^ adapted envelopes may exhibit severe deficiencies in entry fitness and shielding early in their evolution. Considering this, adaptation to CD4^low^ targets may preferentially occur in a sheltered and immune-privileged environment such as the CNS to allow fitness restoring compensatory mutations to occur.

## Author summary

Untreated HIV-1 infection leads to a gradual depletion of CD4^+^ T cells forcing the virus to continuously adapt to an ever-decreasing number of suitable target cells. Our data based on experiments in a highly controlled *in vitro* setting describe one avenue of envelope adaptation that allows the virus to infect target cells expressing low amounts of CD4 such as macrophages. Analysis of the functional envelope phenotypes associated with adaptation of the virus to infect target cells expressing low levels of CD4 highlighted altered entry kinetics of the virus. These alterations resulted in extended exposure of CD4-induced neutralization sensitive epitopes in combination with an increased sensitivity to neutralizing antibodies targeting the CD4 binding site and the V3 loop. Interestingly, a similar phenotype was observed for macrophage-tropic HIV-1 isolates derived from the central nervous system (CNS) of infected individuals raising the possibility that the immune-privileged CNS may favor the development of macrophage-tropic / low CD4 level utilizing envelope variants.

## Introduction

The infection cycle of HIV-1 is intimately linked with the CD4 receptor on target cells. Entry is initiated by the binding of the viral envelope glycoprotein gp120 to CD4, necessitating a high conservation of the CD4 binding site (CD4bs) on the viral envelope [1]. At the same time, the virus faces a humoral immune response targeting the CD4bs [1–4] and disease progression decreases the pool of available CD4 expressing target cells [5–8]. During disease progression multiple forces are therefore acting on the envelope glycoprotein and its interplay with CD4. How these factors shape envelope functional adaptation, and which combination of selective forces is responsible for giving rise to viral phenotypes observed at late disease stages remains unclear. A particular conundrum is the capacity of HIV-1 to maintain high level virus production at late disease stages, even when the classical target cells, CD4^+^ T cells, are heavily depleted [9–12]. Because of this, it was suggested for some time that HIV-1 resolves to replicate in other cell types at later stages [13, 14], which can be linked with use of alternative coreceptors (reviewed in [15–17]). Differential receptor usage most commonly includes varying the capacity of Env to bind CD4 or CCR5, and switching coreceptor use to CXCR4. All of these phenotypes have been observed in late disease states in vivo [18–22].

HIV-1 enters host cells by first binding to CD4 [23, 24] via the gp120 surface glycoprotein subunit [25]. CD4 binding triggers conformational changes in gp120 that expose the co-receptor binding sites to attach to either of the two main co-receptors; CCR5 (R5) [26, 27] or CXCR4 (X4) [28]. The dynamics of CD4 and CCR5 and/or CXCR4 use are important determinants of cellular tropism and transmission of HIV-1. Receptors are expressed independently in various combinations and at different levels on a multitude of human cells, rendering specific cell types differentially susceptible to specific envelope variants [22, 29, 30]. R5 tropic envelopes almost exclusively establish infection [31–34] and allow for the infection of activated effector memory CD4^+^ T cells [35], macrophages, and dendritic cells [36]. A switch in co-receptor use, from R5 to X4 tropism, is well documented at later disease stages, has been observed in 20–50% of patients [37–41] and described in non-human primates (NHP) [42, 43]. CXCR4 usage results in an expansion of cellular tropism of the virus to include naïve CD45RA^+^ CD4^+^ T-cells, which lack CCR5 expression [36], and has often been associated with disease progression [32, 44–47]. Likewise, increased replicative fitness of R5 viruses in later stages of infection has also been linked with rapid disease progression [44, 48–54] though this is not universal [55].

During the course of infection, the HIV-1 envelope must adapt to facilitate replication despite a decrease in its preferred CD4^+^ target cell population. Acute infection rapidly depletes the activated CD4^+^ T cells expressing high CCR5 co-receptor levels abundant in the GALT (gut-associated-lymphoid-tissue) and the genital mucosa [10, 56]. In addition to the death of infected cells, the dramatic reduction in total CD4^+^ T cell counts observed in HIV-1 infection is considered mainly due to apoptotic and bystander cell death [57–60] and possibly killing of CD4^+^ T cells following abortive infection with HIV via pyroptosis [7, 12].

In the progression of untreated infection, the virus thus needs to expand its cellular tropism by gaining access to different tissue compartments and adapting to utilize suboptimal receptor levels and different receptors in humans [61, 62] and NHP models [42, 43]. This is well documented by studies on viruses derived from the central nervous system (CNS). Envelopes from virus circulating in this compartment often display elevated macrophage tropism that has mainly been attributed to an improved affinity for CD4. Paired with almost unanimous R5-usage, increased CD4 affinity allows the virus to infect macrophages expressing low levels of CD4 [20, 21, 63–68]. Macrophage tropism has also been attributed to an increased affinity for CCR5 [20, 69–71]. Consequently, at late disease stages the ability to use low levels of CD4 and R5 or X4, or dual (R5X4) tropism, can provide these envelopes with a broad cellular tropism including macrophages, T cells, DCs and even microglia [21, 36, 63, 66, 69, 72–74].

Thus, the co-evolutionary arms race between the immune system and virus, in combination with the ever-decreasing availability of target cells, produces a complex composition of possible envelope-receptor tropisms specific to disease stages and/or body compartments. Numerous studies have phenotypically characterized envelopes displaying altered receptor- and cellular- tropisms. The impressive body of previous work has almost exclusively focused on identifying associated phenotypes of, and specific mutations associated with, macrophage-tropism derived during infection *in vivo*. The envelopes used in the literature have been isolated from patient material and as such have developed *in vivo* under an undefined collection of selective forces. One of the foremost phenotypes consistently associated with macrophage tropism is the ability to use low levels of CD4. Our study explores the impact that a CD4^low^ selective force has on the resulting envelope phenotype when applied in isolation to address the question whether low CD4 availability by itself is sufficient to generate macrophage tropism.

In this study, we specifically explored the impact of a target cell environment low in available CD4 receptor numbers (CD4^low^), as this may gain in importance at late disease stages during HIV-1 infection. Utilizing an R5 virus isolated from a chronically infected individual, we exposed it to an artificially induced CD4^low^ PBMC environment using a CD4 D1-domain binding DARPin that blocks gp120 binding to CD4 [75]. We show that adaptation of the HIV-1 envelope glycoprotein to CD4^low^ PBMC is sufficient to produce a phenotype similar to that observed in the CNS *in vivo*, displaying high sensitivity to CD4 and neutralizing antibodies as well as increased macrophage tropism. Adaptation to CD4^low^ PBMC resulted in evolution of envelope variants with a higher affinity to CD4 but decreased fitness. Envelopes with a CD4^low^ adapted phenotype suffer reduced particle infectivity, prolonged entry step transitions and increased neutralization sensitivity. We lastly extend the observation of long entry step transitions to several extensively described CNS-derived macrophage-tropic Envs [64, 76–79]. In combination these observations highlight that the emergence of CD4^low^ using viruses must overcome multiple barriers *in vivo*, likely requiring a range of compensatory mutations to offset the reduced neutralization resistance and entry capacity, providing an explanation as to why virus strains with these properties have been observed before the onset of potent neutralization responses [80] and have often been isolated from the immune-privileged CNS [66, 74, 81].

## Results

### Adapting HIV-1 to utilize target cells expressing low amounts of CD4

**Fig 1:**
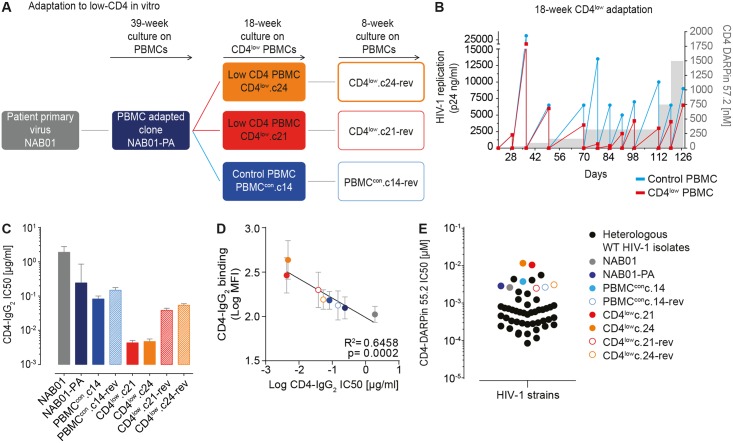
Directed evolution of HIV-1 to utilize CD4^low^ target cells. Adaptation of HIV-1 to CD4^low^ targets *in vitro* (A, B). **(A)** Overview of stepwise adaptation to CD4^low^ PBMC targets and PBMC reversion cultures with normal CD4 levels and the derived envelope clones. **(B)** Summary of the 18 week adaptation to low CD4 expressing target cells. Stepwise decrease in available cell surface CD4 on PBMC was achieved by dose escalation of the CD4 inhibitor DARPin 57.2 (right axis, grey shaded areas). HIV-1 replication as measured by p24 antigen production in culture supernatant on CD4 inhibitor treated cells (CD4^low^ culture, red) and control culture (untreated PBMC, blue) are shown. **(C)** Adaptation to CD4^low^ targets increases sensitivity to CD4-IgG_2_ (PRO542). Mean neutralization sensitivity (IC50) of envelope-pseudotyped viruses on TZM-bl cells derived from two to seven independent assays (error bars = SD) are shown. **(D)** High sensitivity to CD4-IgG_2_ is paired with high binding capacity of CD4-IgG_2_ to Env trimer. Simple linear regression analysis of CD4-IgG_2_ inhibitory capacity (IC50 values shown in panel C) and binding of CD4-IgG_2_ to the envelope of the indicated viruses expressed on 293-T cells. Mean fluorescence intensity = MFI. Data are means of two independent experiments; error bars = SD. **(E)** Adaptation to CD4^low^ target cells results in high resistance to CD4 inhibitor compared to wild type HIV-1 isolates. Comparison of IC50 of CD4-blocking DARPin 55.2 against 41 wild-type HIV-1 strains from different clades (black dots, see S3 Table for details on virus panel and individual IC50 values) and the CD4^low^ adaptation virus panel (colored dots, see legend) probed by Env pseudovirus infection on TZM-bl cells. Data are means of one to three independent experiments.

In the present study we were interested to follow the evolution of the HIV-1 envelope protein when confronted with the selective pressure of a cell environment with low CD4 receptor availability. The starting point of our analysis was a subtype B, R5-tropic virus isolate called NAB01, derived during chronic infection [82, 83]. The process of adaptation of this virus to low CD4 expressing target cells and the chronology of clones generated to form an envelope evolution panel for later phenotypic and genotypic characterization is outlined in Fig 1A.

The NAB01 primary isolate was first adapted to replication on PBMC in the absence of neutralizing antibody pressure to allow later dissection of sequence alterations due solely to adaptation to CD4^low^ levels [84]. After 39 weeks of adaptation to PBMC culture, the envelope gene of the NAB01 PBMC-adapted (PA) virus, termed NAB01-PA, was cloned, and inserted into the replication competent TN6 HIV vector backbone [85] to produce the Env-chimeric virus NAB01-PA-TN6 as a control in a previous study [84]. The NAB01-PA Env clone carried culture adaptation mutations found previously in independent NAB01 long-term culture viruses (S2 Fig and [84]). Our *in vitro* target cell setup with low CD4 availability was designed to mimic a CD4^low^ target cell environment in an immunological sanctuary site that is not, or only sub-optimally, reached by neutralizing antibodies, as occurs in the CNS. Employing the NAB01-PA envelope pre-adapted to growth in PBMC *in vitro* in absence of an autologous neutralization response allowed us to study the virus envelope evolution in response to alteration of the CD4 availability in the target cell environment in the following steps.

To mimic a CD4^low^ environment, we cultured NAB01-PA-TN6 on PBMC in the presence of the CD4 inhibitor DARPin 57.2 which competitively interferes with gp120-CD4 binding and thus limits the availability of CD4 on PBMC without interfering otherwise with the cells [75]. Virus was cultured over 18 weeks in the presence of increasing concentrations of DARPin 57.2 or in absence of the inhibitor. The concentration of DARPin 57.2 was incrementally increased during the cultivation period from 15 nM at the start, to a final concentration of 1500 nM after 18 weeks of culture (Fig 1B). Functional envelope clones capable of free-virus infection were isolated from both the CD4-DARPin treated and control culture supernatants (S2 Table). Two unique envelopes, referred to as CD4^low^.c21 and CD4^low^.c24, were chosen for further analysis. CD4^low^.c24 represented the dominant emerged variant representing the bulk sequence of the CD4 DARPin treated culture, whereas CD4^low^.c21 differed from this main sequence in several positions (S2 Table). Two functional envelopes were isolated from the corresponding PBMC control culture and, one PBMC^con^.c14, with high similarity to the bulk sequence and thus representing the main variant, was selected for further characterization (S2 Table).

We next probed if the adaptation to low CD4 levels on target cells results in a stable virus phenotype or if the envelope reverts to wild type once reintroduced into a high CD4 expressing environment. The selected envelope clones of the adaptation and control cultures (CD4^low^.c21, CD4^low^.c24, and PBMC^con^.c14) were re-cloned into the TN6 vector and cultured independently for eight weeks on PBMC in the absence of CD4 inhibitors (termed reversion culture). Functional envelopes representing the main variants were cloned from each reversion culture, and referred to as CD4^low^.c21-rev, CD4^low^.c24-rev, and PBMC^con^.c14-rev (S2 Table) and used to create Env pseudoviruses and Env chimeric TN6 viruses. In sum we compiled a panel of eight envelopes derived from the original patient isolate NAB01 which we refer to as the CD4^low^ adaptation panel, that include the wild type NAB01 Env, the culture adapted control Envs (NAB01-PA, PBMC^con^.c14, PBMC^con^.c14-rev), the CD4^low^ adapted Envs (CD4^low^.c21 and CD4^low^.c24), and reversion Envs (CD4^low^.c21-rev and CD4^low^.c24-rev).

### Affinity to CD4 dramatically increases with adaptation to CD4^low^ T cells

To explore if the adaptation to a CD4^low^ environment changed the virus’s interaction with CD4, we first compared the sensitivity of our CD4^low^ adaptation panel to the tetrameric fusion protein CD4-IgG_2_, also known as Pro-542 [86] (Fig 1C). As high sensitivity to CD4-IgG_2_ denotes a high affinity of the HIV-1 envelope for CD4 [78], changes in sensitivity allowed us to directly monitor a functional impact of the adaptation to lower CD4 levels. *In vitro* culture adaptation to PBMC is known to result in an increased sensitivity to CD4 based inhibitors [87–89]. In line with this, we observed a 7.9-fold increase in sensitivity to CD4-IgG_2_ for NAB01-PA compared to the patient isolated NAB01. The descendant culture adapted clones PBMC^con^.c14 and PBMC^con^.c14-rev only slightly increased their sensitivity further (2.9-fold and 1.6-fold compared to wildtype, respectively), highlighting that NAB01-PA was optimally adapted to *in vitro* PBMC replication (Fig 1C). In contrast, CD4^low^ pressure resulted in a dramatic increase in sensitivity to CD4 inhibition for both CD4^low^.c21 and CD4^low^.c24 with IC50 values 446.3- and 407- fold lower than wildtype (56.5- and 51.5-fold increased sensitivity relative to NAB01-PA), respectively, confirming that these viruses have increased their affinity to CD4 (Fig 1C). However, this high affinity to CD4 was not maintained upon re-exposure to a high CD4 target cell environment on untreated PBMC. Both viruses partially reverted and lost 8.9- and 11.5- fold sensitivity to CD4-IgG_2_ compared to the CD4^low^ clones, for reversion culture clones CD4^low^.c21-rev and CD4^low^.c24-rev, respectively. This suggested a possible fitness deficit associated with the ability to use low levels of CD4.

To further define the viruses’ affinity for CD4 we compared the binding of CD4-IgG_2_ to cell surface-expressed envelope trimers by flow cytometry. The geometric mean fluorescence of cell-bound CD4-IgG_2_ inversely correlated with CD4-IgG_2_ neutralization activity (R^2^ = 0.6458, p = 0.0002; Fig 1D, simple linear regression) confirming that the CD4^low^ adapted strains, which portray high sensitivity to soluble CD4 neutralization, do indeed have an increased affinity for CD4. In line with this heightened affinity for CD4, the CD4^low^ viruses were the least sensitive to CD4-inhibition compared to 41 wild type HIV-1 strains from multiple subtypes (Fig 1E and S3 Table).

### CD4 and CCR5 levels influence differential infection capacity of CD4^low^ adapted strains

To elucidate the impact of the altered interaction with the CD4 receptor, we next analyzed how CD4^low^ adapted viruses infect target cells with variable CD4 and CCR5 receptor expression. To this end we utilized the 293-T cell based HIV-1 receptor affinity profiling system (293 Affinofiles) to express 42 unique combinations of CD4 and CCR5 densities (S1 Fig) as previously described [90, 91]. This matrix of receptor expression levels covers relevant *in vivo* CD4 levels and further offers the opportunity to differentiate the influence of CD4 and CCR5 levels independently of each other. In the un-induced stage, Affinofile cells have been shown to express CD4 at 0.7 antibody binding sites (ABS) / μm^2^) and at maximal induction 64 CD4 ABS/μm^2^ and therefore to reflect the range of CD4 level distribution observed *in vivo* on monocytes (3.1 CD4 ABS/μm^2^), monocyte derived macrophages (3.4 CD4 ABS/μm^2^) and CD4^+^ T cells (78 CD4 ABS/μm^2^) [21]. We infected induced matrices of Affinofiles and analyzed the 42 independent conditions by 3-D surface plots (S2 Fig).

**Fig 2:**
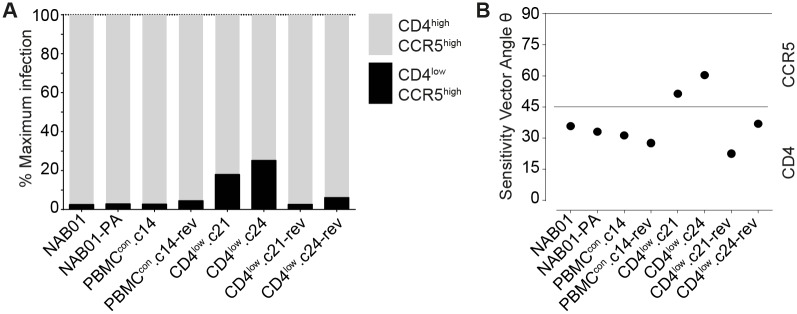
CD4^low^ adapted envelopes infect a wider range of target cells with differential CD4 and/or CCR5 densities. Infection profiles of 293T-Affinofile cells infected with the CD4^low^ panel envelope-pseudotyped viruses. Affinofiles were induced to express a matrix of 42 unique combinations of CD4 and CCR5 and the resulting 3D infection profiles were normalized relative to each envelope’s own maximum infection (S1 and S2 Figs). **(A)** Percent of maximum (high CD4 and high CCR5) level infection retained on cells expressing high CCR5 (2.5 μM Ponasterone A) and the lowest amounts of CD4 (0 μg/ml Doxycycline). Data are from two independent induction and infection assays (S1 and S2 Figs). **(B)** The VERSA sensitivity vector metrics were calculated by fitting a plane to the 3D surface plots (S2 Fig) as previously described by Johnston et al., 2009, and the resulting vector angle indicates a preferential response to changes in CD4 (angles towards 0°) or CCR5 (angles towards 90°). Vector angles from one of two infection experiments are shown.

To assess the efficacy of infection at different receptor densities, we calculated the relative infectivity compared to the maximum activity a given Env reached on Affinofiles for each clone at each Affinofile matrix condition combination (S1 Fig). This analysis highlighted the differential response to decreasing receptor density exhibited by the CD4^low^ adapted Envs in comparison with the rest of the panel (Fig 2). Whereas parental and CD4^high^ target exposed Envs retained only marginal infectivity on targets with low CD4 levels (3% of max for NAB01, NAB01-PA, and PBMC^con^.c14, 5% for PBMC^con^.c14-rev), CD4^low^ viruses were markedly higher (18% and 25% for CD4^low^.c21 and CD4^low^.c24, respectively) (Fig 2A).

Employing sensitivity vector analysis for Affinofile data [91], we estimated the relative sensitivity of each envelope to changes in CCR5 and CD4 levels. In this analysis, sensitivity vector angles 0°≤ θ <45° indicate a higher sensitivity to changes in CD4 level, while angles between 45°< θ ≤90° reflect higher sensitivity to CCR5, and 45° is thereby equal sensitivity to both receptors. In line with an improved capacity to interact with CD4, the CD4^low^ viruses proved to be less steered by changes in CD4 than by CCR5 (57.1° and 60.4° vector for CD4^low^.c21 and CD4^low^.c24, respectively; Fig 2B). Comparing the sensitivity vector angles to the parental and control viruses (33.3°-37.0°) and CD4^low^-reversion clones (26.8° and 36.9° for CD4^low^.c21-rev and CD4^low^.c24-rev, respectively) (Fig 2B), further indicates that the receptor dependency acquired by adaptation to CD4^low^ targets is unfavorable and not stable, as upon replication in a normal CD4 T cell environment, the phenotype is rapidly lost. Control envelopes yielded highly similar values in the vector angle analysis (33.3°-37.0°) suggesting that an optimal dependency for the NAB01 envelope background lies in this range. Of note, the Affinofile sensitivity vector angle was able to elucidate subtle differences in the phenotypes of CD4^low^.c21-rev and CD4^low^.c24-rev. The reversion clones displayed differential avenues of compensation for the apparently unfit CD4^low^ adaptation once returned to unrestricted activated PBMC CD4 levels; CD4^low^.c21-rev showed a markedly lower infectivity compared to CD4^low^.c24-rev (S2 Fig) and it reverted to even higher dependency on CD4 and lower sensitivity to CCR5 (vector angle 26.8°) than the control viruses (33.3°-37.0°; Fig 2B). In contrast, CD4^low^.c24-rev recovered infectivity, exhibiting similar levels of infection to the controls on high CD4 cells, and also developed the highest infectivity of the panel on cells with low CD4. These data suggest that CD4^low^ envelopes have adopted a high tolerance for low levels of CD4, with an associated increased utilization of CCR5. CNS macrophage-tropic Env infection of Affinofiles reported similar effects, namely decreased dependence on CD4, altered interaction with CCR5 (compared to paired non-macrophage-tropic envs)[92, 93].

### Adaptation to CD4^low^ targets results in marked infectivity loss during free virus infection but not in cell-cell transmission and cell-fusion

**Fig 3:**
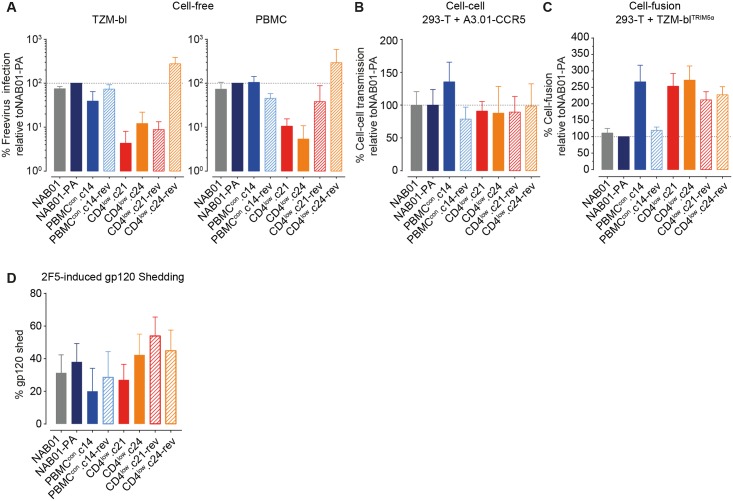
Adaptation to CD4^low^ targets reduces free virus infectivity despite high fusogenicity. **(A)** Cell-free virus infectivity is reduced upon adaptation to CD4^low^ targets. Infectivity of Env-pseudotyped cell-free virus stocks was assessed by titration on TZM-bl (left) and PBMC (right). Infectivity per unit of p24 capsid was calculated (RLU/ng p24) (S3 Fig) and data expressed as percent infection relative to the parental clone NAB01-PA. Data represent the mean of two to three independent TZM-bl titrations, and the mean of three independent experiments on PBMC using different donor batches of three-way stimulated PBMCs and freshly produced virus stocks. Error bars depict standard deviation (SD). **(B)** CD4^low^ adapted viruses maintain infectivity during cell-cell transmission. Env and NLinGluc cell-cell transmission reporter expressing 293-T were co-cultured with A3.01-CCR5 cells in the absence of polycation to measure cell to cell transmission capability of the individual envelopes (S3 Fig) as described [95]. Data shown is the mean of two independent assays, error bars are SD. **(C)** CD4^low^ adapted viruses have high cell-fusion efficacy: Env and NL-Luc-AM reporter expressing 293-T cells were co-cultured with rhesus Trim5α-expressing TZM-bl target cells to measure fusogenicity of panel envelopes. Data shown are the means of three independent assays, error bars are SD. **(D)** CD4^low^ adapted viruses are not prone to shedding of gp120. Gp120 shedding from Envelope-pseudoviruses in response to treatment with the MPER nAb was assessed as the percentage of gp120 content after 2F5 treatment relative to mock-treated controls normalized to p24 input. Data shown are the means of three independent assays, error bars are SD.

We next measured the capacity of the CD4^low^ virus panel to infect target cells with different ranges of CD4 and CCR5 levels using single round replicating Env pseudo-viruses (Fig 3A). We first probed infection on TZM-bl, which were previously estimated to carry 4x10^5^ CD4 and 1.3x10^4^ CCR5 receptors per cell [94], and stimulated PBMC which were estimated to express a pre-activation average of 6x10^3^ CD4 and 593 CCR5 receptors per cell [29]. In line with the reported receptor densities, we observed higher absolute infectivity on TZM-bl than PBMC (S3 Fig). NAB01, NAB01-PA, and the culture controls PBMC^con^.c14, and PBMC^con^.c14-rev, showed infectivity within a 3.6-fold range on both TZM-bl and PBMC (Fig 3). In contrast, adaptation to CD4^low^ targets led to a 2.5–24.8 fold and 3.3–13.3 fold loss in infectivity on TZM-bl and PBMC compared to the parental clone NAB01-PA for CD4^low^.c21 and CD4^low^.c24, respectively. Notably, infectivity was restored in only one of the reversion culture clones: CD4^low^.c24-rev (Fig 3A).

We next explored whether the CD4^low^ adaptation may have led to improved spread in culture via cell-cell transmission to compensate for the attenuated free virus infectivity observed for CD4^low^.c21 and CD4^low^.c24 (Fig 3A). Therefore, we probed the ability of the virus panel to infect via cell-cell transmission (Fig 3B) and to undergo cell-cell fusion (Fig 3C). Cell-cell transmission capacity was similar across the entire virus panel (Fig 3B). The control Env PBMC^con^.c14 proved to be the most efficient envelope in cell-cell transmission. Most importantly however, we detected no pronounced deficiency for the CD4^low^ adapted strains CD4^low^.c21 and CD4^low^.c24, which reached an average of 91% and 88% of the infectivity of NAB01-PA, respectively. This suggested that cell-cell transmission may aid the virus to overcome fitness deficiencies when altering receptor usage. Cell-fusion, however, portrayed an entirely different picture (Fig 3C). The CD4^low^ adapted clones CD4^low^.c21 and CD4^low^.c24 together with the control culture envelope PBMC^con^.c14 were the most effective in initiating fusion, reaching 267%, 253%, and 272% of NAB01-PA, respectively (Fig 3C). Thus, the free virions of the CD4^low^ adapted strains fail to efficiently infect despite intact, if not improved fusogenicity of the Envs.

Decreased free virus infectivity of the CD4^low^ adapted Envs could potentially indicate a low stability of the Env trimers, i.e. trimers that are prone to shed gp120. While Env expressed on the surface of an infected cell is continuously replenished by newly expressed Env, a low stability would affect cell-cell transmission less than free virus infection, where Env on viral particles degrades over time without active replacement. This would fit with the observed pattern of retained cell-cell transmission and low free virus infection. We thus examined the propensity of the Env panel to gp120 shedding in response to the MPER-specific bnAb 2F5, a potent inducer of gp120 shedding [96]. While CD4 induced shedding would also be interesting to define in the context of HIV-1 entry, the large differences in CD4 affinity of the panel nearing a three-log variation in CD4 sensitivity (Fig 1) would not allow for a direct comparison of effects. Measuring 2F5 induced gp120 shedding we observed a modest shedding activity against the CD4^low^ Env clones (27% and 42% for CD4^low^.c21 and CD4^low^.c24, respectively) which was in the range of shedding observed for the parental (NAB01) and control (CD4^low^.c14) envelopes (31% and 20%, respectively). Hence, a high propensity to gp120 shedding cannot be underlying cause of the decreases free-virus infectivity of CD4^low^ compared to parental and control Env clones.

### Adaptation to CD4^low^ targets results in infectivity gain on macrophages

High affinity to CD4 has been associated previously with the capacity to infect cells expressing low levels of CD4, in particular macrophages [18, 66, 78, 79, 97, 98]. To test macrophage infectivity we produced two types of differentially conditioned monocyte derived macrophages known to vary in CD4 and CCR5 expression [29], M-MDM have previously been shown to express an average of 125 CD4 and 55 CCR5 receptors per cell while G-MDM have lower levels of both receptors with an estimated 50 CD4 and 15 CCR5 per cell [29]. We verified the relative CD4 expression on our cell preparations and indeed observed 3.1 fold lower CD4 levels on G-MDM (S4 Fig).

**Fig 4:**
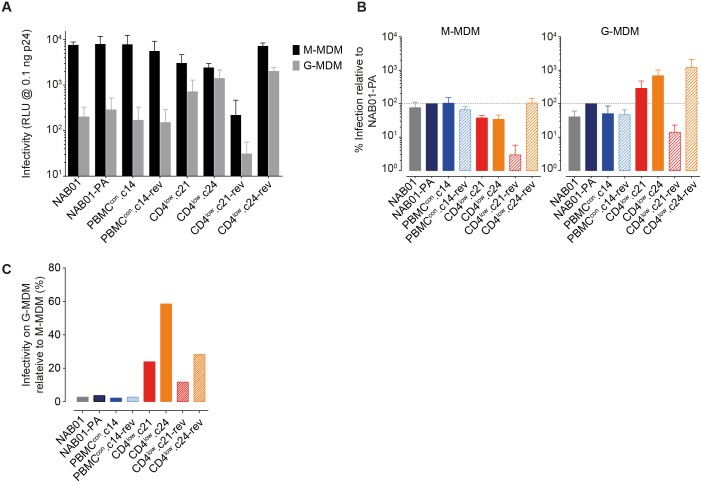
Adaptation to CD4^low^ allows efficient infection of macrophages. **(A)** Two types of macrophages, M-MDM and G-MDM expressing differential CD4 levels (S4A Fig) were infected with 0.1ng p24 of ultra-centrifuged Env-pseudotyped luciferase reporter virus stocks infection measured on day seven post-infection by quantifying luciferase reporter activity (relative light units (RLU)). Data are means from two individual donors, input of ultracentrifugation purified virus was standardized by p24 content, error bars = SD. **(B)** Infection of M-MDM and G-MDM by CD4^low^ adapted viruses relative NAB01-PA (data derived from A. **(C)** Comparison of M-MDM and G-MDM infectivity. Shown is the relative infectivity of G-MDM compared to M-MDM infection (data derived from A).

As HIV-1 infection of MDM can show high donor variability we verified that the trend observed in Fig 4 was maintained in eight donor cell batches (S4 Fig). Of note, absolute infectivity of ultra-centrifuged Env pseudovirus on M-MDM normalized to p24 content of stocks was close to what we observed for PBMC (S3 and S4 Figs). Overall, G-MDM infection was markedly lower than M-MDM infection (27.4 fold lower NAB01-PA infection on G-MDM than M-MDM; Fig 4A) in line with the lower expression of entry receptors on G-MDM cells.

Interestingly, free-virus infection of M-MDM with the CD4^low^ adapted viruses yielded infection levels closer to parental virus and controls on M-MDM (Fig 4A and 4B) than on PBMC or TZM-bl (Fig 3A) with M-MDM infectivity of CD4^low^.c21 and CD4^low^.c24 being only 2.6 and 3.0 fold lower compared to NAB01-PA on, respectively (Fig 4B). The pattern of G-MDM infection was strikingly different. While absolute G-MDM infection was generally lower for all probed viruses (Fig 4A), the CD4^low^ clones infected G-MDM with higher efficiency than the parental and culture control viruses (2.5 fold and 4.9 fold higher compared to NAB01-PA, respectively; Fig 4B). CD4^low^.c21 and CD4^low^.c24 reached 23.9% and 58.5%, respectively, of M-MDM infection levels on G-MDM (Fig 4C). In contrast, the parental clone NAB01 showed only 2.7% infectivity on G-MDM compared to M-MDM, similar to NAB01-PA and PBMC^con^.c14 (3.6% and 2.2%, respectively). Infection of both M-MDM and G-MDM again emphasized the contrasting phenotypes of the two reversion clones. CD4^low^.c21-rev was the least and CD4^low^.c24-rev one of the most effective of the entire panel in infection of both MDM subtypes. In sum, the differential infectivity of macrophage subtypes across virus strains supported the observations made using the Affinofile system. Differential macrophage infections further highlighted that adaptation to usage of low CD4 levels can optimize infection of specific cell subsets and thus needs to be considered as an important parameter in shaping target cell tropism throughout disease progression.

### Adaptation to low CD4 levels gives rise to envelope mutations associated with macrophage tropism

**Fig 5:**
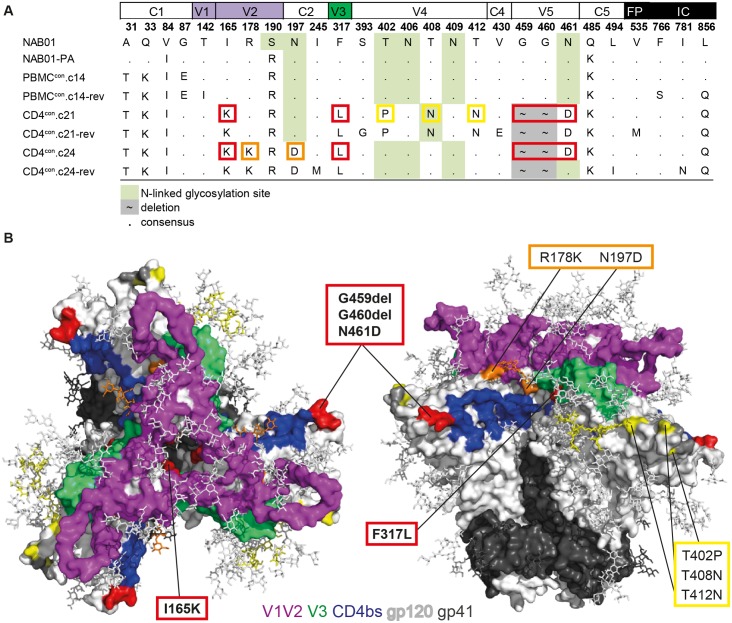
Gp120 sequence mutation pattern following adaptation to CD4^low^ targets. **(A)** Summary of amino acid mutations acquired as a result of adaptation to long term *in vitro* culture and adaptation to CD4^low^. Green shading indicates mutations affecting N-linked glycosylation sites. Red boxes denote mutations that occur in both CD4^low^ adapted clones, yellow and orange boxes indicate mutations that occurred only in CD4^low^.c21 and CD4^low^.c24, respectively. **(B)** Structural representations of mutated residues in NAB01 associated with adaptation to low levels of CD4 mapped onto crystal structure 5fyj of X1193.c2 SOSIP [100]. Dark gray residue shading indicates limits of non-resolved region of gp120 V4 loop. Missing from the model are the residue at 402, within the non-resolved region of V4, and the glycans at residues 408 and 461 which are not present on this subtype G Env variant. Structure rendered using PyMol version 1.4.1 [101].

We next explored the sequence alterations that occurred during adaptation to CD4^low^ pressure (Fig 5 and S4 Table). Locating globally relevant sites of macrophage tropism determination has proven difficult [21]. Numerous mutations have been associated with macrophage tropism but were largely found to be strain-specific. Of note, the parental virus NAB01 and all variants derived through culturing lack E153G, T283N, or N386D signatures of enhanced macrophage tropism (reviewed in [14]). CD4^low^ adaptation resulted in five mutations in gp120 common to both CD4^low^.c21 and CD4^low^.c24 clones (Fig 5A and S4 Table). The five mutations observed in CD4^low^ Envs include I165K in the V2-loop, F317L in the V3 loop, and in the V5 region a dual deletion at G459 and G460 in combination with N461D that eliminates a potential N-linked glycan at position 461 in the CD4 contact site in V5 [99]. The location of the mutated residues is highlighted on the crystal structure model of subtype G Env trimer X1193.c2 [100] as this structure provides a high resolution of the V4 region (Fig 5B). Although this Env is missing glycans that we found to be under selection pressure in our adapted viruses, overall mapping of the mutated residues onto the X1193.c2 structure provided interesting insights on their approximate spatial distribution.

#### V2 loop

The mutation I165K is located within the V2 loop in the trimer association area at the apex of the trimer (Fig 5B) and is present in only 0.04% of 4907 envelope sequences analyzed from the Los Alamos Database (S5 Fig). The sequences in this database are dominated by virus derived from plasma and PBMC, and thus are less likely to capture frequencies amongst macrophage or CNS replicating viruses. However, the I165K mutation is found in only 1 of the 98 patients with subtype B Env sequences with CNS ontology included in the HIVbrainseqDB [102], further suggesting that this mutation is rare. In addition to I165K, the V1V2 net charge increased upon adaptation to PBMC from -0.2 to +0.8 and further to +1.8 upon CD4^low^ adaptation. This level of V1V2 charge was sustained for both reversion clones (S4 Table).

#### V3 loop

No overall net changes in charge of the V3 were observed (S4 Table). The V3-loop mutation F317L that emerged in the CD4^low^ clones is also rare (0.14% of the Los Alamos HIV sequences, 0.06% of subtype B; S5 Fig). F317L has previously been associated with the CD4^low^ adaptation of CNS-derived envelopes [79], and is also prevalent in 14 of 98 patients with CNS derived Subtype B viruses from the HIVbrainseqDB [102]. Residue 317 is located within the highly conserved hydrophobic tip of the V3 loop and has previously been associated with heterodimer stability [103].

#### CD4bs

The CD4^low^ adapted envelopes shared three mutations proximal to the CD4bs; two deletions in the V5 (G459del, and G460del) and an N461D mutation which eliminates a highly conserved N-linked glycosylation site (found in 99.86% of 4907 sequences in the Los Alamos Database) (S5 Fig). Of the three mutations that the CD4^low^ envelopes shared, N461D was the only one which reverted upon passaging on PBMC with normal CD4 levels in one of the isolated reversion clones (CD4^low^.c24-rev).

#### N-glycosylation sites

Both CD4^low^ clones lost further glycosylation sites present in the parental strain (Fig 5A). Mutations T402P, T408N, and T412N present in CD4^low^.c21 and CD4^low^.c21-rev are located in a highly variable domain of the V4. These mutations resulted in loss of glycans at positions 402 and 409, and a transfer of an N-linked glycosylation site from 406 to 408. Removal of glycans at 410–412 has been reported to decrease infectivity and increase the potential of the envelope to generate neutralizing antibodies, presumably by exposing neutralization sensitive epitopes [104]. Likewise, N197D, present in both CD4^low^.c24 and CD4^low^.c24-rev, destroys a potential N-linked glycosylation site at the apex of the trimer spike known to modulate sensitivity to CD4bs nAbs [105], cause increased CCR5 binding [106, 107] and, in combination with V/T200 (found in all the NAB01 derived clones), has been associated with macrophage tropism in the CNS [108].

Interestingly, by acquiring a L856Q mutation the CD4^low^ adapted clones loose the gp41 C-terminal di-leucine internalization motif thought to play a role in reducing Env content from infected cell surface membranes [109].

### High affinity to CD4 is associated with prolonged transitioning during entry from CD4-bound stage to CCR5 engagement and fusion

Altered conformational transitions of the trimer upon receptor engagement have been suggested as an attribute of macrophage-tropic envelopes able to use low levels of CD4 [98]. Considering their increased ability to accomplish membrane fusion (Fig 3C) and to utilize low levels of CD4 to infect (Figs 2A, 2C and 4), we hypothesized that CD4^low^ adaptation may have an influence on the kinetics of attachment and entry.

**Fig 6:**
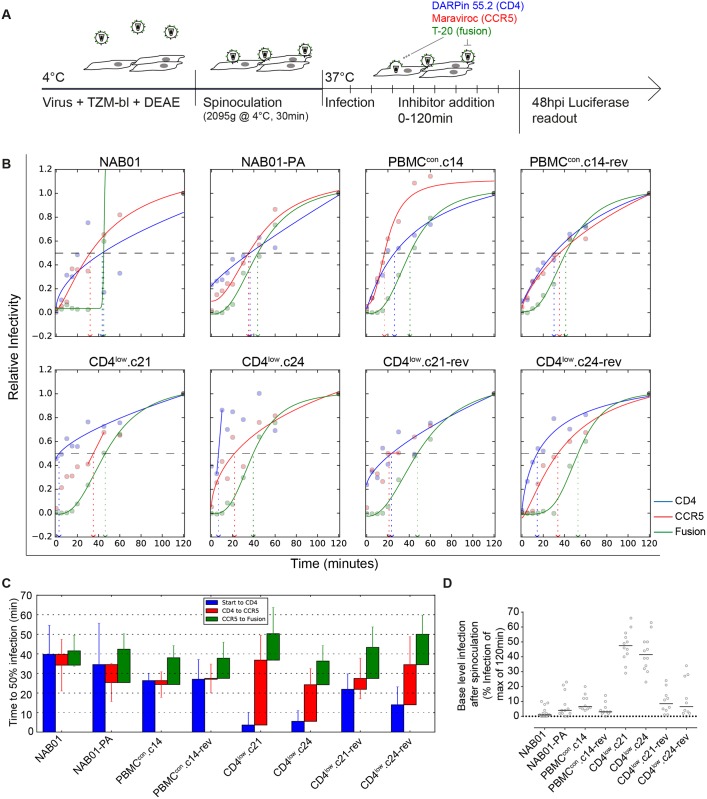
CD4^low^ adapted viruses need extended time to transition between steps in the entry process. **(A)** Schematic of entry kinetic assay to measure timing of CD4 binding, CCR5 binding, and fusion. Virus is added to TZM-bl in the presence of the polycation DEAE, spinoculated onto cells at 2095g for 30 min at 4°C to limit conformational changes upon CD4 binding. Infection is synchronized by the addition of warmed media and inhibitors targeting CD4 (DARPin 55.2), CCR5 (Maraviroc), and fusion (T-20) added in saturating concentrations at 0, 5, 10, 15, 20, 30, 45, 60, and 120 min post start of infection. **(B)** For each envelope, one representative time course of infection is shown. Infectivity data are normalized to infection at 120min post infection and all treatment conditions are shown as relative infectivity compared to this 100% level. **(C)** Definition of transition times required to reach 50% of transition to CD4 bound, CCR5 bound stage and fusion. For each inhibitor and each of at least eight replicate measurements derived from four to six independent experiments, T½ values of infection times were estimated. The mean of these estimates is a proxy for the time required to reach 50% CD4 resistance, 50% CCR5 resistance, and 50% fusion resistance. Error bars denote SD. **(D)** Percentage of viruses already resistant to CD4 blocking following the 30 minute spinoculation at 4°C. Data points are derived from four to six independent experiments done in replicates. Horizontal bars depict means.

To assess the relative timing of transitions between the three key steps in the entry process—CD4 engagement, coreceptor binding and fusion—we employed a time-course inhibitor addition experiment with inhibitors targeting CD4 (CD4-DARPin 55.2), CCR5 (maraviroc) and HIV-1 fusion (T-20) (Fig 6A). In this assay setup, synchronized infection is achieved through spinoculation at 4°C (a temperature which prevents receptor engagement) and a shift to 37°C post-spinoculation to initiate entry. Saturating concentrations of inhibitors were added to replicate infection wells at progressive time points from the initiation of infection (0 min) up to 120 min post infection (Fig 6A). For each inhibitor, we considered the relative infectivity compared to the 120 min post infection value and fitted infection curves to estimate the time required by each virus to reach 50% of entry level reached by the 120 min treatment point (Fig 6B).

When comparing the kinetic of the parental NAB01 virus with the two CD4^low^ adapted clones, we observed a striking difference of the times needed to transition from the CD4 bound to the CCR5 bound state as well as from CCR5-to-Fusion (Fig 6C). Whereas the time required to reach 50% fusion differed only 1.4-fold across all eight panel viruses (36.3–50.3 minutes post infection), the time required to complete 50% CD4 binding and 50% CCR5 engagement differed markedly. Most strikingly, we found that the CD4^low^ adapted envelopes, though they engaged CD4 rapidly, required a significant increase in time for the transition between CD4 binding and CCR5 engagement (Mann-Whitney test, p = 0.00003 and 0.00024 for comparison between NAB01 and CD4^low^.c21, NAB01 and CD4^low^.c24, respectively). While time windows for CD4 and CCR5 binding tightly overlapped for NAB01 and the derived PBMC adapted strains, suggesting a very rapid transition between CD4 and CCR5 engagement for these strains, the CD4^low^.c21 and CD4^low^.c24 strains had a time window of 33.2 and 19.6 min between CD4 and CCR5 engagement, respectively. This gap proved largely due to a very rapid initial engagement of CD4 (Fig 6D) and not a postponing of CCR5 binding. In line with their high affinity for CD4, the CD4^low^ adapted envelopes already established a firm CD4 binding during the 30 minute spinoculation at 4°C, reaching a mean of > 40% of the maximal infection (CD4^low^.c21 at 47.4% and CD4^low^.c24 at 42.4%; Fig 6C). This was in striking contrast to the rest of the NAB01 virus panel that only reached infection levels between 2.7% and 8.5% before initiation of the entry process by shifting cultures to 37°C. Interestingly, rapid CD4 binding appeared to be an unfavorable trait that the virus only maintained under selection pressure likely as it requires a more open conformation of the trimer. Both reversion clones increased the time to CD4 engagement, and consequently shortened the CD4 to CCR5 transition window.

**Fig 7:**
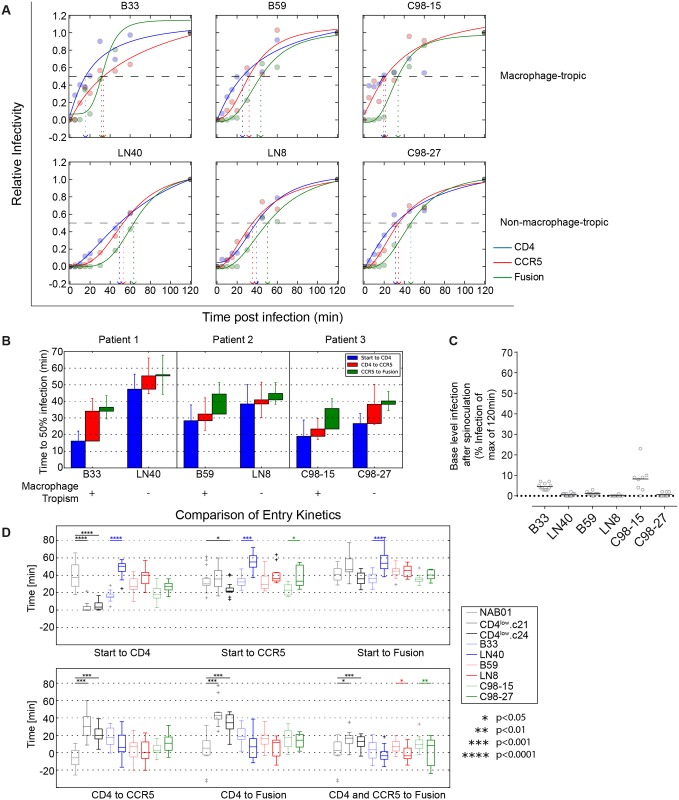
CNS-derived Macrophage-tropic viruses show similar entry pattern with rapid engagement of CD4. **(A)** to **(C)** Times to reach 50% resistance to CD4, 50% CCR5, and fusion inhibitors was determined for the shown pairs of patient derived macrophage-tropic and non-macrophage-tropic viruses as described in Fig 6B to 6D. Data for each inhibitor and virus combination were derived from at least eight replicates from four to six independent experiments. **(A)** For each envelope, one representative time course of infection is shown, normalized to infection at 120min post infection. Data shown is for a replicate representative of the calculated mean of all replicates. **(B)** Time intervals between four stages of the entry process (synchronized start, CD4 binding, CCR5 attachment, fusion) were compared by Mann-Whitney tests of NAB01 and CD4low viruses and M-tropic and non-M tropic pairs from the analyzed three patients. Only envelopes from the same patient (same principal color) were compared. **(C)** Data depict the percent of virus already resistant to CD4 blocking following the 30min spinoculation at 4°C. Individual data points are two replicates from each of four to six independent experiments. Horizontal bars depict means. **(D)** Statistical analysis of entry kinetics. Data points from four to six individual experiments were combined before fitting the curves and averaging individual T½ values. Estimated time intervals between the four stages of the entry process (synchronized start, CD4 binding, CCR5 attachment, fusion) were compared by Mann-Whitney tests. Only envelopes from the same patient (same principal color) were compared. Alternate statistical analysis using paired replicates before curve fitting shown in S6 Fig.

We then asked whether the phenotype we observed in the CD4^low^ envelope clones could potentially be found *in vivo*, particularly in the CNS where the immune pressure by neutralizing antibodies is less active. To address this question we probed the entry kinetics of a panel of three well established macrophage-tropic envelopes from the CNS (B33 and B59), and plasma (C98-15) together with patient-matched non-macrophage-tropic Envs from lymph nodes (LN40, LN8), and plasma (C98-27) (S5 Table) [64, 76–79]. The rapid engagement of CD4 exhibited by the CD4^low^ Envs was reproduced by the macrophage-tropic envelopes (Fig 7A and 7C). The time to CD4 binding for all three patient pairs (B33/LN40, B59/LN8, C98-15/ C98-27) shows a trend of faster CD4 engagement by the macrophage-tropic envelopes when compared to their non-macrophage-tropic paired Env (Fig 7B), though this difference achieves statistical significance only between B33 and LN40 (Fig 7D). Of all patient derived envelopes, the brain-derived B33 also displays the longest CD4 to CCR5 transition (Fig 7B and 7D). The phenotype we observed for CD4^low^ Envs may therefore occur *in vivo*, and in particular in the CNS, where antibody pressure is commonly reduced compared to other compartments. All transitions of the entry process were significantly different between the CD4^low^ clones and NAB01, except time from start to fusion (Fig 7D, Mann-Whitney test). The trends highlighted by the more extreme phenotype of the CD4^low^ envelopes were displayed most similarly by B33, and by B59 and C98-15 to a lesser degree (Fig 7D). To test the sensitivity of our analysis method, we estimated T_½_ values in additional ways. First, we averaged the data points from all replicates, prior to fitting only one curve, and since two replicates were always conducted within the same experiment, we also pooled these two data points before fitting the curves and averaging individual T½ values. Whenever taking the average, we also considered using the median instead of the mean. These different data treatment and analysis methods mostly decreased the power to detect significant differences (S6 Fig), however they had only a minor impact on estimated parameters (S7 Table) and subsequently the trends observed between macrophage-tropic/CD4^low^ compared to non-macrophage-tropic/NAB01 were reproducible.

**Fig 8:**
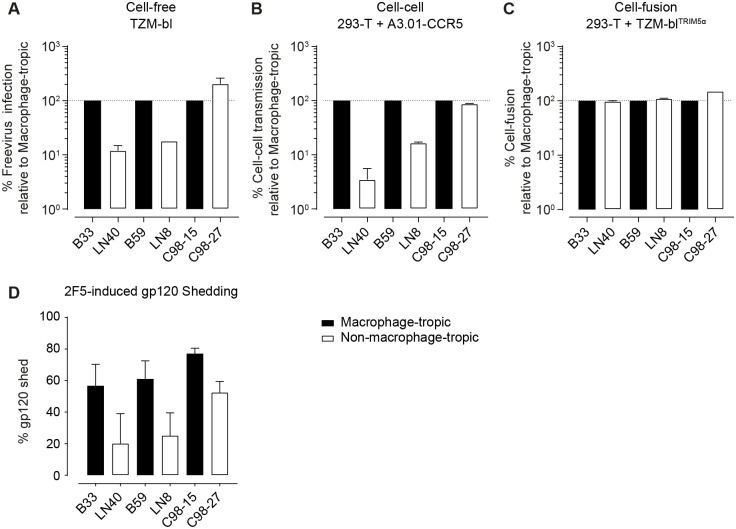
CNS-derived Macrophage-tropic viruses show increased infection capacity and gp120 shedding. **(A)** Cell-free virus infectivity is increased in CNS-derived macrophage tropic Envs. Infectivity of Env-pseudotyped cell-free virus stocks was assessed by titration on TZM-bl. Infectivity per unit of p24 capsid was calculated (RLU/ng p24) (S7 Fig) and data expressed as percent infection relative to the patient-paired non-macrophage-tropic Env. Data shown is the mean of two independent assays, error bars are SD. **(B)** CNS-derived macrophage-tropic Envs have improved infectivity during cell-cell transmission. Env and NLinGluc cell-cell transmission reporter expressing 293-T were co-cultured with A3.01-CCR5 cells in the absence of polycation to measure cell to cell transmission capability of the individual envelopes (S7 Fig) as described [95]. Data shown is the mean of two independent assays, error bars are SD. **(C)** CNS-derived macrophage-tropic Envs maintain similar cell-fusion efficacy to non-macrophage tropic Envs: Env and NL-Luc-AM reporter expressing 293-T cells were co-cultured with rhesus Trim5α-expressing TZM-bl target cells to measure fusogenicity of panel envelopes. Data shown is the mean of two independent assays, error bars are SD. **(D)** CNS-derived macrophage-tropic viruses have increased shedding of gp120. Envelope-pseudoviruses carrying mouse CD4 were treated with 2F5 to induce gp120 shedding and immobilized using magnetic beads. Shed gp120 and non-bound virus was washed away and gp120 and p24 levels measured by ELISA as described [96]. The difference between gp120 levels of 2F5 treated and to mock-treated controls is depicted as % gp120 shed. Data shown are the means of three independent assays, error bars are SD.

We further profiled the patient paired macrophage-tropic and non-tropic Env functionality in free-virus infectivity, cell-cell transmission, and cell-fusion to determine the depth of phenotypic similarity shared between CNS-macrophage tropic and CD4^low^ Envs. Interestingly, the infectivity of the CNS-derived macrophage-tropic B33 and B59, but not the plasma-derived C98-15 macrophage-tropic Env was increased relative to their respective non-macrophage tropic control Envs (Fig 8A). The lymph-node derived LN40 and LN8 reached only 12% and 18% of B33 and B59’s free-virus infectivity, while C98-15 had half the infectivity of the non-macrophage tropic variant C98-27 (205%). A similar trend was observed in cell-cell transmission where LN40 and LN8 reached 3.5% and 16.5% of the cell-cell capacity of B33 and B59, respectively (Fig 8B). The capacity of all three macrophage-tropic Envs to initiate cell-fusion was similar to the controls, ranging from 98% to 150%, relative to non-macrophage tropic (Fig 8C). Of particular note, all three macrophage-tropic Envs showed an increase in gp120 shedding relative to their paired non-macrophage-tropic Envs, regardless of the tissue of origin (Fig 8D). Both B33 (57%) and B59 (61%) lost more than twice as much gp120 as LN40 (20%) and LN8 (25%). The plasma-derived Envs were more similar, showing 77% and 52% gp120 shedding for C98-15 and C98-27, respectively.

### Prolonged transitioning to CCR5 engagement coincides with increased vulnerability to V3 loop and CD4i directed antibodies

**Fig 9:**
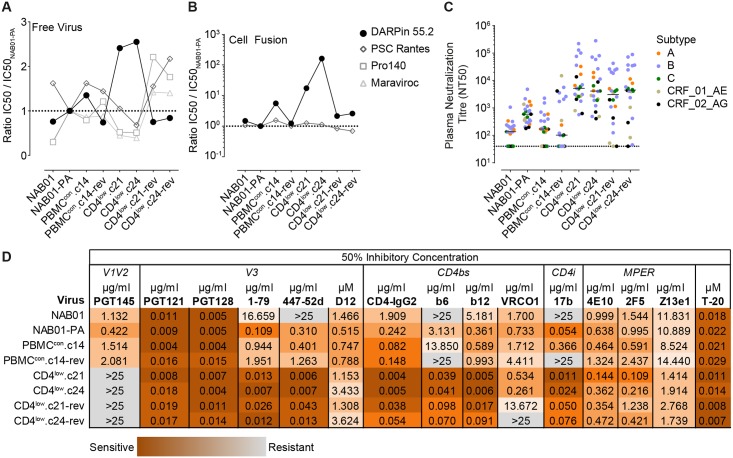
CD4^low^ adapted envelopes show heightened sensitivity to inhibitors targeting the CD4bs, V3 loop, and CD4i epitopes, and patient plasma. **(A)** and **(B)** Sensitivity of the CD4^low^ viruses to inhibitors of CD4 (DARPin 55.2) and CCR5 (PSC RANTES, Pro140, Maraviroc). IC50 values are shown relative to the IC50 of NAB01-PA in **(A)** free virus entry on TZM-bl and **(B)** 293T-TZMbl^TRIM5α^ cell fusion. Individual IC50 values are listed in S6 Table. **(C)** Sensitivity of CD4^low^ virus panel to heterologous plasma neutralization. Data are medians derived from neutralization titer on TZM-bl cells of patient plasmas from 24 individuals with different HIV-1 subtype chronic infections (eleven subtype B, four subtype A, and three of each subtype C, 01_AE, and 02_AG). **(D)** Sensitivity of CD4^low^ virus panel to neutralizing antibodies and Env targeting inhibitors (S1 Table). IC50 values were derived in a standard pseudovirus neutralization assay on TZM-bl cells. Darker shading indicates higher sensitivity. Data shown in A, B, and D are mean values from at least two independent assays for each inhibitor.

We next examined the sensitivity of the virus panel to entry inhibitors and neutralizing antibodies (nAbs) targeting diverse regions of the envelope (S1 Table) to elucidate the consequences of adaptation to low levels of CD4 for shielding and the susceptibility to neutralizing antibodies. Analysis of the sensitivity of free virus infection to anti-CD4 and CCR5 receptor agents using NAB01-PA as the point of reference highlighted that a modest decrease in sensitivity to CD4 inhibition in the CD4^low^ strains was mirrored by an equally modest increase in sensitivity to the CCR5 inhibitors (Fig 9A and S6 Table). This agrees with the higher dependency on CCR5 observed in the Affinofile analysis for the CD4^low^ Envs (Fig 2 and S1 and S2 Figs) and was also supported by the observation that the reversion clones lost CCR5 inhibitor sensitivity while regaining sensitivity to CD4 inhibition. Interestingly, the CD4^low^ strains portrayed a much higher resistance to CD4 inhibition during cell-cell fusion while sensitivity to CCR5 inhibition showed no alteration (Fig 9B).

Adaptation of primary isolates to growth *in vitro* in the absence of neutralizing antibody pressure commonly leads to the emergence of virus variants with increased neutralization sensitivity [87]. This was also true for the PBMC long-term cultured NAB01-PA, which displayed higher sensitivity to chronic patient plasma from 24 individuals with chronic HIV-1 infection (eleven subtype B, four subtype A, and three of each subtype C, 01_AE, and 02_AG) (Fig 9C), and nAbs targeting V3, CD4bs, and CD4i (Fig 9D) than the parental NAB01. Sensitivity to V3 glycan and MPER nAbs, and the fusion inhibitor T-20 did not differ between NAB01 and NAB01-PA. In contrast, the V2 glycan nAb PGT145, which depends on a closed trimer conformation for neutralization, showed a 2.7-fold reduced activity against NAB01-PA. CD4^low^ adaptation amplified this phenotype and resulted in a substantial increase in neutralization sensitivity to nAbs targeting the CD4bs, CD4i, and V3 epitopes, as well as patient plasma (Fig 9C and 9D), ranging from 446-fold (for nAb b6) to 4301-fold (for nAb 447-52d). The V3 targeting DARPin D12 [110], which recognizes the V3 loop in a structure-dependent manner, slightly decreased in activity against the CD4^low^ and reversion strains compared to NAB01-PA. Similarly, the conformational epitope of PG145 was lost completely in the CD4^low^ envelopes, and was not restored in the reversion clones. Reversion culture viruses also only showed a partial recovery of resistance to V3 and CD4bs nAbs.

### Influence of CD4^low^ adaptation on entry stoichiometry

**Fig 10:**
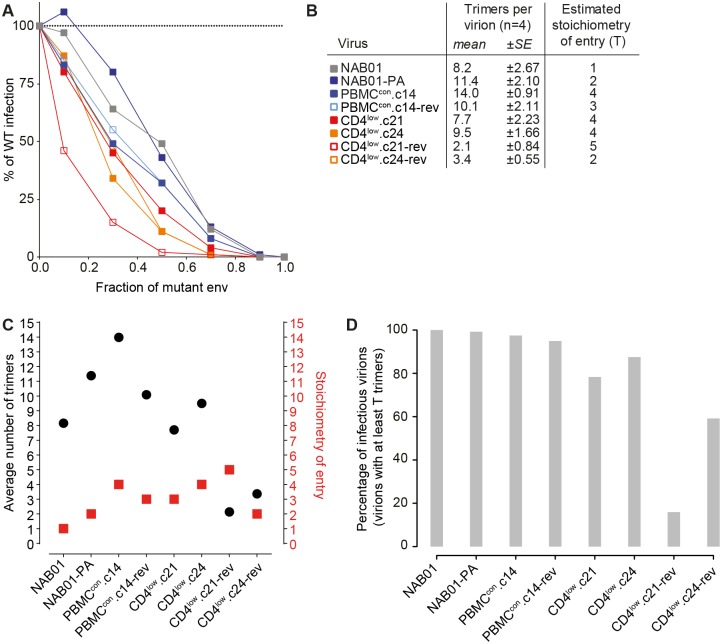
Envelopes adapted to low levels of CD4 require a higher proportion of their available trimers to complete entry. **(A)** Relative infectivity of mixed trimer infection experiments with CD4^low^ panel viruses using the R508S/R511S dominant-negative Env mutants. Infectivity of pseudotyped virus stocks expressing the indicated ratios of wild type and dominant-negative mutant Envs was measured on TZM-bl reporter cells. Infectivity of virus stocks containing solely the respective WT envelope were set as 100%. Data depict mean from two independent experiments. **(B)** Experimentally defined mean trimer number per virion measured from four independent assays (S8 Table) were used to derive mathematical estimates of the entry stoichiometry T based on data shown in (A) as described [112]. **(C)** Graphical presentation of mean trimer number per virion and estimated stoichiometry of entry as shown in (B). **(D)** The percentage of infectious virions, i.e. virions with at least T trimers, was calculated for each single viral variant based on trimer numbers distributed according to a discretized B-distribution with the measured mean (Fig 10B) as described in [114].

Considering the substantial changes in neutralization sensitivity, entry kinetics, and infectivity across the virus panel, we were next interested to explore if the stoichiometry of entry is altered. It is plausible that the Env conformations which favor CD4 binding that are induced by *in vitro* culture in the absence of neutralization pressure, and adaptation to low levels of CD4 are associated with energetic losses, and in consequence may need more trimers to complete entry [111–113]. To test this, we generated dominant negative Env mutants by introducing R508S/R511S (SEKS) to all panel viruses to knock out the furin-like protease cleavage site between gp120 and gp41, as described [112]. Mixed trimer virus preparations with SEKS variants in varying ratios with wild-type Env were generated and analyzed for infectivity (Fig 10A). Using this data and the average number of trimers per virus particle that was determined in parallel (Fig 10B and S8 Table) allowed us to estimate the minimal number of trimers required for viral entry (T) using a previously established mathematical model [111–113]. While the primary virus NAB01 required only one trimer for entry (T = 1), adaptation to PBMC in vitro culture caused an increase of minimal number of trimers required for entry ranging from T = 2 to T = 4. CD4^low^ adaptation maintained T = 4, underlining a continued need for more trimers to be employed in the entry process for these virus variants. The two CD4^low^ reversion clones showed a substantial decrease in the average trimer number per virion, decreasing by 3.7- and 2.8- fold for CD4^low^.c21-rev and CD4^low^.c24-rev from their parental clones, respectively. For CD4^low^.c21-rev this was particularly striking as the average number of trimers per virion (2.1) was lower than the estimated number of trimers required for entry (T = 5). Thus only a small fraction of CD4^low^.c21-rev virions will carry the required number of trimers necessary to facilitate entry (Fig 10D) and this provides a potential explanation for the particularly low infectivity observed across cell types for (Figs 3A and 4B).

## Discussion

During disease progression HIV-1 must overcome the decreasing supply of activated CD4^+^ T cells, which express high levels of CD4 and the CCR5 coreceptor [10, 35, 115, 116]. The transition of the virus to altered receptor usage and the ensuing changes in cell tropism at later disease stages have been extensively studied over the past 30 years, yet many details remain elusive [14, 20, 36, 65, 69–71, 117–120]. The observed transitions during the course of the infection are thought to be needed to allow the virus to infect a broader range of host cells. This may require altered coreceptor usage [15, 77, 121], or modifications to allow infection of cells that express lower CD4 levels. Low CD4 usage in particular is exemplified by R5 viruses exhibiting macrophage tropism [63, 65–67, 74, 77, 78, 98, 122–124]. Development of increased CD4 binding capacity by gp120 has been implicated in high macrophage tropism [125–127], which may have relevant consequences in disease progression (reviewed [128]), however the forces that lead to this phenotype have not been clearly defined. One clue has been provided by the frequent association of highly macrophage-tropic envelopes with CNS infection in late disease [129] suggesting features of this compartment particularly favor or facilitate the development of envelopes with high CD4 affinity. Adaptation of the HIV-1 envelope to CD4^low^ conditions warrants study to elucidate potential intermediate evolutionary states, CD4^low^ associated phenotypes, and to improve our understanding of the forces driving development of this niche phenotype at the high end of the continuum of CD4 use.

In the current study we adapted an R5-tropic HIV-1 envelope, isolated from a chronic patient, to CD4^low^ conditions on PBMC *in vitro*. Our setup mimicked the environment that is thought to occur in early infection and the CNS compartment, which is with little or no neutralizing antibody pressure. The ensuing CD4^low^ adapted envelopes displayed a very high affinity binding to CD4. Altered CD4 affinity specifically opens transmission to a new population of cells via an amplified ability to use low amounts of CD4 on the target cells. While these characteristics suggest an overall benefit to CD4^low^ adaptation, we lay out here that this comes at severe costs for the virus in terms of general infectivity and vulnerability to neutralization.

Adaptation of the NAB01 parental WT envelope to *in vitro* PBMC infection resulted in the S190R and V84I mutations. Herschhorn and colleagues have recently described the impact of mutating the highly conserved leucine at position 190 in the V2 loop [130]. Replacing the L with either an alanine or arginine provided JR-FL (subtype B) and BG505 (subtype A) with improved macrophage infection, increased sensitivity to CD4 binding, and higher neutralization sensitivity by non-neutralizing Abs. Their evidence suggests the mutations enrich the amount of envelopes present in a functional state between the ‘closed’ wild-type conformation and the CD4-bound ‘open’ conformation. While NAB01-PA only showed slightly improved macrophage infection, sensitivity to non-neutralizing Abs 1–79, 447-52d, 17b, and b6 were markedly increased, in agreement with an enrichment of the in-between state of envelope conformation.

CD4^low^ adaptation in the NAB01 envelope background occurred by the removal of several structural elements that presumably relaxed restrictions of access for both CD4 and CD4bs specific nAbs. Particularly notable were a deletion of glycosylation sites and part of the V5 loop (affecting residues 459–461) projecting into the space leading to the CD4bs (Fig 5B) [99]. While these changes were linked with an increased ability to bind CD4, the loss of shielding resulted in increased accessibility for CD4bs and CD4i nAbs that are normally well shielded off as exemplified by the increase in efficacy of the CD4bs mAb b6 and the CD4i mAb 17b (Fig 9D). Addition of a positive charge at residue I165K, which comes into close proximity with the neighboring subunits at the trimer association region, has the potential to reduce shielding, by disrupting the interplay of the neighboring V1V2 regions. Mutation of the conserved F317 residue in the V3 is linked with decreased association of gp120 and gp41 [103], further suggesting a decreased conformational stability for the Envs adapted to CD4^low^.

Our detailed analysis of CD4^low^ envelope entry kinetics further supports a reduction in the energy provided by conformational rearrangements during the multi-step entry process. Following a very rapid CD4 engagement, the time required to transition between steps in the entry process is significantly extended for CD4^low^ adapted envelopes (Figs 6 and 7 and S6 Fig), yet interestingly, the overall length of entry remained comparable. The delay within entry occurs most drastically between binding of CD4 and attachment to CCR5 in the CD4^low^ clones. Given the ability of CD4^low^ Envs to bind to CD4 rapidly and at temperatures normally restrictive to conformational changes, it is surprising that CCR5 attachment does not proceed any quicker, indicating that the CD4^low^ viruses depend on the CCR5 interaction to release the required amount of energy to progression to the next stage of entry. We speculated this may relate to a decreased potential energy carried within the open conformation of the trimers, and could prospectively impact entry stoichiometry. A requirement for a larger number of trimers in order to overcome opposing membrane potentials during entry could help explain the observed delay between CD4 and CCR5 binding. However, the T (stoichiometry of entry) values estimated for CD4^low^ envelopes were comparable to the non-CD4^low^ envelopes adapted to *in vitro* culture, both types requiring four trimers for entry. Notably, the CD4^low^ adapted clones showed a higher T paired with lower overall trimer content on virions. Thus a large fraction of viruses in these populations will not carry the minimal amount of trimers necessary for entry and a high proportion of the available trimers needs to be engaged in the entry process to make infection possible likely explaining the low infectability of these viruses. Whether the CD4^low^ viruses have a CD4 bound conformation that differs from wild type requiring longer to interact with CCR5 or whether this indicates that more CCR5 receptor interactions per trimer have to occur will be further interesting possibilities to explore in future studies.

The *in vitro* adaptation to CD4^low^ targets generated envelope variants with the likely beneficial phenotype of expanded cellular and receptor tropism and drastically increased CD4 binding. However, as mentioned above, the beneficial phenotype comes at the cost of reduced infectivity (Fig 3 and S3 Fig), extended exposure of neutralization sensitive epitopes during entry (Figs 6 and 7), and increased neutralization sensitivity (Fig 9C and 9D). Partial reversion of these phenotypes after readapting to CD4^high^ conditions indicates that an increase in CD4 binding affinity may result in an ultimately less-fit envelope.

The CD4^low^ envelopes proved generally neutralization sensitive in line with the reduced shielding and a prolonged exposure of neutralization sensitive epitopes during entry. It may therefore be critical for this phenotype to develop away from the pressure provided by nAbs *in vivo*. Our examination of CD4^low^ linked phenotypes suggests possible mechanisms that could support development of the neutralization sensitive phenotype. One avenue of adaptation is suggested by our finding that free virus infectivity is dampened for CD4^low^ envelopes but their ability to disseminate via cell-cell transmission is comparable to the primary isolated Env (Fig 3). Maintenance of the cell-cell pathway could potentially serve as a rescue mode of viral transmission supporting previous observations [95, 131, 132]. Cell-cell transmission could thereby allow transmission of less fit virus while adaptation passes through neutralization sensitive intermediates on the way to a more optimally fit phenotype adapted to a CD4^low^ environment. The *in vitro* passaging protocol used in this study is expected to encourage the selection of Envs competent in free-virus infection. Passaging was performed for 16 of the 18 weeks with virus supernatant only. The observed conservation of cell-cell capacity in the face of cell-free selective pressure suggests either a strong impetus to maintain this phenotype, or a lack of effect of the CD4^low^ adaptation and passaging on the cell-cell phenotype. In addition, we expect that the absence of neutralization pressure during *in vitro* passaging allowed the reversion Envs CD4^low^.c21-rev and CD4^low^.c24-rev remaining sensitive to neutralization.

In the cell-cell experiment we used one T cell line, A3.01-CCR5 [131], as targets which, as all T cells, has high CD4 levels and is thus comparable to TZM-bl cells in respect to CD4 expression. However, while our data show that a large variation exists in the CD4 use and infectivity of our envelope panel (Figs 1C–1E, 2, 3A and 4), the cell-cell transmission shows little to no difference across the panel. While we cannot rule out that differences in cell-cell transmission may occur when target cells with lower levels of CD4 are involved, in a comparison of CD4^high^ expressing targets only free virus transmission was affected.

In conjunction with competency in cell-cell transmission, the fusion capacity of CD4^low^ envelopes is 2-fold higher than that of NAB01, which may pose a problem to the development of CD4^low^ use *in vivo*. Primarily, cell-fusion does not lead to productive infection and syncytia resulting from cell-fusion may not be long-lived [133]. Therefore, increased fusion in combination with higher CD4 use could result in a dead end path for the virus and thus be a further reason why the virus rarely opts for high CD4 affinity *in vivo*.

The extreme CD4^low^ phenotype was lost during re-adaptation to high CD4 expressing targets even in the absence of nAb pressure. Adaptation to CD4^low^ may therefore encounter resistance from the various fitness related requirements of virus replication *in vivo*. This is supported by the establishment of novel infections by non-macrophage-tropic R5 envelopes [134] due to the fitness costs associated with CD4^low^ use described here, and the bottleneck at transmission selecting high-fitness variants in newly established infections [135]. We have addressed the question of how CD4^low^ envelopes might develop *in vivo* by comparing the entry phenotypes with those of well-defined CD4^low^ using envelopes isolated from patients to find that the CD4^low^ phenotype can be recapitulated amongst viruses replicating in the CNS. It is intriguing that one of the CNS derived envelopes (B33) displayed a phenotype similar to the CD4^low^ adapted clones 21 and 24, while the other (B59) did not. A potential explanation for this discrepancy could be that B33 and B59 were isolated from CNS at different stages of CNS infection [136] and/or replicated within different cell types [93]. An additional non-competing possibility is that the status of the blood-brain-barrier is affected by the progression of infection [137] differentially in the respective patients, hence allowing more neutralizing antibodies, and/or plasma derived viruses to traverse to the CNS in certain patients. Of the five shared mutations found in both CD4^low^ clones (Fig 5), the only one shared by one of the CNS derived envelopes is 317L which is also found in B33 and has been associated with the ability to use low levels of CD4 [79].

The phenotypic characterization of the CNS-derived macrophage-tropic Envs highlights similarities (e.g. similar entry kinetics) but also differences to our CD4^low^ adapted Envs suggesting that our *in vitro* adapted clones represent an intermediate evolutionary phase with reduced cell-free infectivity. The CNS-derived macrophage-tropic Envs, which displayed superior infectivity in cell-free and cell-cell transmission modes relative to their paired controls (Fig 8), may have passed this stage and acquired compensatory mutations that preserve the entry phenotype but restore infectivity. In particular the elevated infection competency of these CNS-derived Envs despite increased gp120 shedding suggests these Envs have undergone severe selective refinement *in vivo* during their development. It is tempting to speculate that cell-cell transmission in vivo may have supported the evolution of these shedding-prone, yet highly infecting competent envelopes. While our in vitro selection favored free transmission as only supernatant was passaged, in vivo both transmission pathways will be available.

Though the CNS was once considered a site of immunological privilege, it has been established that various branches of the immune system operate within the CNS [138–140] including the production of limited amounts of antibodies from within the cerebrospinal fluid [141]. Envelopes evolving within the CNS may therefore encounter some humoral immune pressure, as B59 may have, though the impact of such an interaction on envelope evolution remains an open question. The lack of an extended entry phenotype in C98-15, isolated originally from plasma, further shows that the phenotype observed is not a universal feature of all highly macrophage-tropic envelopes and may potentially differ depending on whether the clone recently evolved or was circulating (and adapting) for an extended period of time. Nevertheless, the overall trend within each pair of macrophage-tropic and non-macrophage-tropic envelopes mirrors the difference between CD4^low^ adapted and non-adapted Envs from our panel including an increased speed of CD4 binding and extension of transition steps (Fig 7D).

Arrildt and colleagues [125] recently conducted an interesting profiling of phenotypes of primary macrophage-tropic isolates. They found that macrophage-tropic primary isolates were not significantly different from matched control Envs in a variety of functional characteristics including infectivity and entry kinetics, as well as neutralization sensitivity to plasma and V1V2 targeting nAbs. Proposed evolutionary intermediate Envs accordingly showed a moderate sensitivity to CD4. In contrast our CD4^low^ Envs and the CNS-derived macrophage tropic Envs differ from their patient-matched controls. As our Envs, like the CNS-derived macrophage tropic Envs, have not been exposed to humoral immunity during adaptation, it is tempting to speculate that they represent an early adaptive stage that could develop only where humoral immunity is low as in sanctuary sites as the CNS. Infectivity defects, as the CNS derived strains highlight, need not to be associated with this phenotype of CD4^low^ usage and likely are only a transition point in the evolution towards a stable and fit variant.

In summary, adaptation to low levels of CD4 on target cells appears to occur in direct opposition to nAb escape, providing a plausible explanation for the association of highly macrophage-tropic envelopes with the CNS and their appearance before nAb development [80].

Inhibition of the gp120 and CD4 interaction using reagents that bind to the same domain of cellular CD4 that interacts with gp120 to yield effective therapeutics has been investigated extensively [75, 142–145]. Our study, in addition to describing the phenotype resulting from CD4^low^ adaptation, highlights potential routes of escape from CD4 blocking. The envelope adapted to CD4-blocking gains a wider cellular tropism by an increased ability to bind to CD4, raising the possibility that blocking access to CD4 therapeutically could potentially accelerate the generation of envelope variants found normally in late disease stages with increased CD4 binding affinity. The CD4^low^ adapted envelopes generated in our *in vitro* system, as well as one brain-derived envelope (B33) developed high neutralization sensitivity in parallel with CD4 affinity, which suggests for B33 that it may also have developed in the absence of nAb pressure. Intriguingly, a macrophage-tropic isolate derived from plasma (C98-15), which evidently must have been exposed to neutralizing antibodies, retained neutralization resistance despite developing the other features required for macrophage tropism. The same was true for the CNS derived virus B59 opening the possibility that this strain encountered neutralization pressure as well. Both C98-15 and B59 showed a less extreme phenotype in the entry kinetics compared to the CD4^low^ viruses and B33. However, in both cases the same trend in kinetics shift was evident in comparison to non-macrophage-tropic Envs from the same patients (Fig 7). Considering the potential danger of widening the host cell repertoire, administering CD4 derivatives rather than targeting CD4 and the CD4bs directly may be more advisable. That this leads to potent suppression has been shown in the past for CD4-IgG_2_ (aka Pro542; [146, 147]), and small-molecule CD4 mimetics [148–151] and with currently unexcelled potency for eCD4-Ig [152]. However, escape pathways for these compounds also need to be meticulously explored to exclude changes in the host cell repertoire and unfavorable alterations in viral fitness. Thus far only few studies have been dedicated to study escape from CD4 mimicking and soluble compounds [153, 154], with more effort defining escape from CD4bs specific Abs elucidating fitness costs associated with escape [155].

In sum our analysis describes a set of phenotypic features directly associated with CD4^low^ adaptation of one subtype B envelope in the absence of nAb pressure that is consistent with phenotypic changes found in two CNS-derived Envs. We have also connected some of these phenotypes to envelopes of primary patient isolates suggesting that the environment where they evolved *in vivo* potentially shares features with the microenvironment we generated *in vitro*. The phenotypic changes that came alongside the adaptation to use low levels of CD4, in particular the alterations in entry kinetics and stoichiometry, trimer content and infectivity may open means to better understand the limitations that evolution of CNS macrophage tropism faces. Mechanistic studies on larger sets of CNS and peripheral macrophages may thus aid to inform vaccine design towards limiting macrophage tropism, ideally preventing the spread of infection into the CNS.

## Materials and methods

### Ethics statement

Peripheral blood mononuclear cells (PBMC) were purified from buffy coats from anonymous blood donations from healthy individuals obtained by the Zurich Blood Transfusion Service (http://www.zhbsd.ch/) under a protocol approved by the local ethics committee.

Patient plasma from twenty-four individuals with chronic HIV-1 subtype A (N = 4), B (N = 11), C (N = 3), CRF_01_AE (N = 3), or CRF_02_AG (N = 3) infections were obtained from biobank samples previously collected during three approved clinical trials the Swiss Spanish treatment interruption trial (SSITT), the Swiss HIV Cohort study (http://www.shcs.ch) and the Zurich Primary HIV-infection (ZPHI) study (ClinicalTrials.gov identifier NCT00537966) [156–160]. Written informed consent was obtained from all individuals according to the respective studies as stated in the quoted publication according to the guidelines of Canton Zurich and the local ethics committee of all participating clinics.

### Reagents

We thank the following individuals for providing inhibitors, antibodies and antibody expression vectors either directly, or via the NIH AIDS Research and Reference Reagent Program (NIH ARP): W. Olsen (Progenics, Tarrytown, New York, USA) for CD4-IgG_2_ and PRO140; D. Burton (The Scripps Research Institute, La Jolla, California, USA) for b6, b12, PGT121, PGT128, PGT145, Z13eI; J. Mascola (VRC, Bethesda, Maryland, USA) for VRC01; M. Nussenzweig (The Rockefeller University, New York, USA) for 1–79; J. Robinson (Tulane University, New Orleans, USA) for 17b; D. Katinger (Polymun Scientific, Vienna, Austria) for 2G12, 4E10, and 2F5; and Marc Connors (NIC, Bethesda, Maryland, USA) for 10E8. 447-52d was purchased from Polymun Scientific, Vienna, Austria; T-20 from Roche Pharmaceuticals, Basel, Switzerland; and Maraviroc from Pfizer, UK. Human CD4 specific DARPins 55.2 and 57.2 were expressed as described (Schweizer, Rusert et al. 2008 [75]). A detailed list of all inhibitors and antibodies with their specifications can be found in S1 Table.

### Cell lines

293-T cells (American Type Culture Collection (ATCC)) and TZM-bl cells ([161], obtained from the NIH ARP) were cultivated in DMEM with 10% heat inactivated FCS and 1% Penicillin/ Streptomycin. Rhesus Monkey Trim5α expressing TZM-bl cells (TZM-bl^rhTRIM5α^) were generated as described [131]. A3.01-CCR5 cells [131] were maintained in RPMI with 10% heat inactivated FCS and 1% Penicillin/Streptomycin. Affinofile cells [90, 91] were thawed every two months and maintained in DMEM media supplemented with 10% dialyzed fetal bovine serum and 50μg/ml Blasticidin (Invitrogen, Massachusetts, USA).

### Peripheral Blood Mononuclear Cells (PBMC)

Healthy donor PBMC were isolated from buffy coats and stimulated as described [162] and cultivated in RPMI with 10% heat inactivated FCS, 1% Penicillin/Streptomycin and 100 units/ml (U) human recombinant IL-2 (Hoffmann-La Roche, Basel, Switzerland).

### NAB01 and NAB01-PA envelopes and generation of Env-chimeric, replication competent TN6 viruses

The cloning of the CCR5-tropic subtype B envelope NAB-01 (previously described as NAB1pre-cl_39x (GenBank database entry EU023918; (http://www.ncbi.nlm.nih.gov/GenBank/index.html; [84]), which was derived from the virus isolate of a chronic infected individual, patient NAB01 [82], has been previously described [84, 162]. After 39 weeks of adaptation of the NAB01 isolate to PBMC culture, envelope genes of the adapted virus were cloned from culture supernatant. One clone, termed NAB01 PBMC-adapted (PA), was selected for follow up as representative culture adapted clone as it harbored mutations in V84I and S190R, that were previously observed in PBMC adaptation of NAB01 clones ([84] and EF643665, EF643666, EF643668-EF643670, EF643673-EF643675). The Env NAB01-PA was then cloned into the replication competent TN6 HIV vector backbone [85] to produce the Env-chimeric virus NAB01-PA-TN6 [84] which was used as starting point for the CD4^low^ adaptation. Of note, chimeric envelopes inserted into the TN6 backbone receive the signal peptide and first three amino acids of Env from the original TN6 NL4-3 Env.

### Adaptation of NAB01-PA to CD4^low^ expressing target cells

To follow the evolution of NAB01-PA in a low CD4 environment, NAB01-PA-TN6 was passaged on PBMC in the presence of increasing concentrations of a CD4 inhibitor, the high-affinity CD4-binding DARPin 57.2 that efficiently blocks HIV-1 gp120 binding to CD4 [75]. In a parallel control culture, NAB01-PA-TN6 was propagated on the same batches of PBMCs in the absence of the CD4 blocking agent. After 18 weeks of culture and a final maximal concentration of 1.5 μM DARPin 57.2 (Fig 1B), several functional envelope clones capable of free-virus TZM-bl infection were isolated from both the CD4-DARPin treated and control culture supernatants by RT-PCR (S2 Table). Two unique envelopes, CD4^low^.c21 and CD4^low^.c24, representing the main mutation patterns observed, were selected for further follow up (S2 Table). To study whether the phenotype of the derived CD4^low^.c21 and CD4^low^.c24 clones is stable or reverts to wild type once back in a CD4 high environment, CD4^low^.c21, CD4^low^.c24 and PBMC^con^.c14 TN6 chimeras were further cultured for eight weeks on untreated PBMCs in the absence of CD4-binding DARPin. After eight weeks, functional envelope genes were isolated from each of the reversion cultures, and representative clones referred to as CD4^low^.c21-rev, CD4^low^.c24-rev and PBMC^con^.c14-rev (rev = reversion culture) chosen for follow up. All envelopes selected for follow up were cloned into pcDNA3.1 expression vector (Invitrogen, Carlsbad, California, USA) as described [84] to allow phenotypic characterization of cell expressed envelopes and Env-pseudoviruses (see below) and further cloned into the TN6 vector to create replication competent chimera.

### Patient derived paired macrophage and non-macrophage-tropic envelopes

The previously described patient-derived paired macrophage and non-macrophage-tropic envelopes (B33, LN40/B33, B59, LN8, C98-15, and C98-27 [64, 76–79]) were re-cloned from pSVIII plasmids into pcDNA3.1 expressions plasmids by AT-overhang ligation to allow direct comparison with the CD4^low^ virus panel.

### Envelope pseudotyped HIV-1 luciferase reporter virus

Envelope pseudotyped HIV-1 were produced as described [162]. Briefly, 293T cells were transfected with pcDNA3.1 plasmids encoding the respective envelope genes and the Luciferase expressing HIV-1 backbone pNLluc-AM (gift from A.J. Marozsan and J.P. Moore) at a ratio of 1:3 using polyethylenimine (PEI, linear 25 kDa, Polysciences, Eppelheim, Germany). After 8-16h of transfection, medium was replaced with fresh culture medium. Pseudovirus supernatant was collected 48h post transfection and filtered through 20 μm pore micro filters. Filtrates were then either stored at -80°C or ultra-centrifuged at 49’000g and 4°C for 90 minutes and suspended in PBS before being stored at -80C. Envelope-pseudotyped virus expressing mouse CD4 was produced as previously described [96].

### Pseudovirus inhibition assays

Pseudovirus inhibition assays were performed using TZM-bl cells and PBMC as previously described [162, 163]. Briefly, serial dilutions of inhibitors were pre-incubated with target cells or viruses depending on target epitope, for 1hr at 37°C, then virus, cells, and inhibitor were added together and incubated for 48–72 hours. Infection supernatants were then aspirated, cells lysed, luciferase substrate (Promega, Madison Wisconsin, USA) added and the presence of luciferase quantified by measuring relative light units (RLU) using a Dynex MLX 96-well plate reader.

### CD4 binding activity of Env variants

To estimate CD4 binding activity of Env variants, we performed a cell-based envelope binding assay. 293-T cells were transfected with pCMV-rev and envelope encoding pcDNA3.1 plasmids in a 1:4 ratio with polyethyleneimine (PEI, linear 25 kDa, Polysciences, Eppelheim, Germany) and incubated for 36 hours to induce membrane expression of the envelope. CD4 binding activity of cell surface expressed Env was analyzed by flow cytometry using biotin labelled CD4- IgG_2_ and detection with streptavidin-PE (BD, New Jersey, USA).

### Monitoring dependence on receptor levels by infection of Affinofile cells

CD4 and CCR5 dual-expressing 293-T Affinofile cells were induced and infected in a matrix layout as previously described [90, 91]. Briefly, 1.2x10^5^ or 1x10^4^ cells were seeded per well of a 24-well culture plate for quantitative flow cytometry of CD4 and CCR5 expression levels, or 96-well clear-bottom culture plate for infections, respectively, and allowed to adhere overnight before being treated with perpendicular serial dilutions of Doxycycline (Sigma Aldrich, Missouri, USA) and Ponasterone A (Invitrogen, Massachusetts, USA) to induce CD4 and CCR5 expression, respectively. Surface expression levels of CD4 and CCR5 were quantified between 18-24h post induction by quantitative flow cytometry using QuantiBRITE beads and PE-labelled antibodies specific for CD4 (clone Q4120; Sigma Aldrich, Missouri, USA) and CCR5 (clone 2D7; BD Biosciences, New Jersey, USA). Affinofile cells were infected 18-24hrs post-induction using 10^5^ RLU of virus per well (calculated by titration on TZM-bl cells) in the presence of 10 μg/ml Diethylaminoethyl (DEAE; Ammersham plc, UK) and luciferase readout was performed after 48hrs of infection.

### Cell-cell transmission

To specifically measure viral infectivity in cell-cell transmission, we employed a recently described A3.01-CCR5 cell based assay [95]. Briefly, this assay utilizes an NL4-3 derived pseudotyped HIV-1 backbone with an intron-regulated Gaussia luciferase LTR-reporter construct called inGluc which is co-transfected with an Env expression plasmid into 293-T donor cells [13, 16, 164], a kind gift from Dr. M Johnson. The reverse orientation of the reporter and the intron allow luciferase expression only after correct splicing, packaging into viral particles and infection of and expression in the A3.01-CCR5 target cells. Free virus infection was restricted by the omission of DEAE in the infection medium as described previously [131].

To measure infectivity in free virus and cell-cell transmission, 2.5x10^5^ 293-T cells per 6-well were transfected with Env expression plasmid and NLinGluc backbone for cell-cell in a 1:3 ratio, using polyethylenimine (PEI) as transfection reagent. To test cell-cell infectivity, the cells were detached after six hours of incubation and 5x10^3^ cells were seeded in 100 μl per 96 well. 1.5x10^4^ A3.01-CCR5 target cells in 100 μl RPMI medium were added to the 293-T cells per 96 well. After 60 h of incubation at 37°C, Gaussia luciferase activity in the supernatant was quantified using the Renilla Luciferase Assay System (Promega, Madison Wisconsin, USA) according to the manufacturer’s instructions.

### Cell fusion assay

We employed a co-culture of HIV-1 pseudovirus transfected 293-T cells and the LTR-firefly luciferase reporter expressing TZM-bl cells to obtain information on the fusion potential of the probed envelope clones. To limit influence of free virus in the readout, we used TZM-bl cells that express rhesus macaque Trim5α, as expression of rhTrim5α in target cells restricts free virus infection [95, 131]. 293-T cells were PEI transfected for six hours with *env*-pcDNA3.1 and pNL AM GFP backbone. Transfected cells were then detached, washed once at 300g for 3min, suspended in 1200μl culture medium and 100μl were added to 10 replicate wells of a clear-bottom 96 well plate containing 1.5x10^4^ TZM-bl^rhTrim5α^ in the presence or absence of inhibitors. After 24 hours, luciferase activity was recorded.

### Detection of gp120 shedding

Gp120 shedding following treatment with 2F5 was measured as previously described [96]. Envelope-pseudotyped virus carrying mouse CD4 (mCD4) were ultracentrifuged at 49’000g for 90 minutes at 4°C. The virus pellet was resuspended in TBS +2% BSA for 120–200 minutes at room temperature and treated with 100 μg/ml of mAb 2F5 for 18–20 hours at 37°C. Virions were immobilized with magnetic Dynabeads coated with rat mAb L3T4 specific for mCD4 (Thermo Fisher, Massachusetts, USA) using a magnetic tube rack (DynaMag-2, Thermo Fisher, Massachusetts, USA) and washed twice to remove shed gp120 and non-bound virions. Captured viral particles were lysed with TBS +1% Empigen and gp120 and p24 content measured by ELISA. The gp120 content was normalized to p24 content of each sample and shedding was expressed as a percentage of the mock-treated controls.

### HIV-1 infection of primary monocyte-derived-macrophage cultures

Monocytes were purified from freshly isolated healthy donor PBMC using MACS anti-CD14-coated magnetic beads and MACS separation columns (Miltenyi Biotec, Germany). Purity of viable cells ranged between 96–98% as assessed by CD14^+^ staining in flow cytometry (CD14-PE from Life-Technologies; IgG_2_a-PE Isotype from BD Pharmingen; Live-Dead near infra-red viability stain from Life Technologies; pooled mouse serum used to block fc receptors at 20% (gift of L. Hangartner). 5x10^4^ monocytes per well were seeded into 96-well culture plates (Greiner Bio-One GmbH, Germany) for infection experiments, or 2x10^6^ cells per well seeded ultra-low attachment surface 6-well plates (Sigma Aldrich, Missouri, USA) for flow cytometry analysis. Monocytes were differentiated for six days in differentiation medium (RPMI1640 supplemented with 1% Pen/strep, 10% fetal-calf serum, 5% pooled human AB serum (Sigma Aldrich, Missouri, USA), 4mM Glutamax (Thermo Fisher, Massachusetts, USA)) and either 100ng/ml M-CSF or 100ng/ml GM-CSF (Immunotools GmbH, Friesoythe, Germany) to produce two different phenotypes of monocyte-derived-macrophages [165–167], known to express differential levels of CD4 and CCR5 [29]. After six days of differentiation @ 37°C in 80% humidity and 5% CO_2_, differentiation medium was replaced with culture medium (RPMI 1640, 10% FCS, 1% penicillin and streptomycin) and incubated at 37°C overnight. Pseudovirus infections and flow cytometry of surface receptors were performed on day seven post-isolation. Before infecting MDMs, one well of each MDM type was imaged with an Incucyte ZOOM system using a 10x objective lens and ‘scan-on-demand’ with the phase contrast channel. Cell morphology was compared to [166] to confirm cultures were independently differentiated as one phenotype or the other by the presence of ‘fried egg’ morphology for G-MDM, and ‘spindle-cells’ for M-MDM (S4A Fig). Envelope pseudotyped virus supernatants were titrated onto MDMs and allowed to incubate at 37°C in 80% humidity and 5% CO_2_ for seven days. Firefly luciferase production was measured using a Dynex MLX plate reader (high gain setting) and Bright-Glo Luciferase Assay System (Promega, Madison Wisconsin, USA) on day 14 post-isolation (day seven post infection). To analyze surface marker expression, MDM were detached with cell dissociation solution (Sigma Aldrich, Missouri, USA) and stained with CD4-PE (BD, New Jersey, USA), CD195-PE (Biolegend, California, USA, California, USA), CD64-FITC (Biolegend, California, USA), or CD163-BV421 (Biolegend, California, USA) mAbs, or matched isotype controls, to measure expression of HIV receptors CD4 and CCR5(CD195) and macrophage differential phenotypic markers CD64 and CD163 [168](S4A Fig). Pooled mouse serum (gift of L. Hangartner) was added at 20% to all staining solutions to block unspecific binding of mouse mAbs to MDM Fc receptors.

### Envelope sequencing and analysis

Cloned envelope genes were sequenced on an ABI 3130xl genetic analyzer (Applied Biosystems, Rotkreuz, Switzerland) as described [84]. Gp160 protein characteristics were calculated using ProtCalc for charge (http://protcalc.sourceforge.net/) and the NIH N-GlycoSite tool (http://www.hiv.lanl.gov/content/sequence/GLYCOSITE/glycosite.html) to count potential glycosylation sites. Mutation prevalence in the Los Alamos National Laboratory HIV sequence database was performed using QuickAlign (https://www.hiv.lanl.gov/content/sequence/QUICK_ALIGNv2/QuickAlign.html) web alignment with all 4907 complete sequences available at the time of query. Subtype and Tissue ontogeny- filtered sequences were downloaded from the HIVbrainseqDB [102] and the prevalence of specific mutations queried using a custom algorithm, available on request. Envelope gene sequences of all clones were deposited in the GenBank database entries KX673206-KX673212.

### Entry kinetics

Time of inhibitor addition experiments were performed to define entry kinetics with small modifications to previously described protocols [112, 169]. Briefly, 6x10^3^ TZM-bl cells in DMEM supplemented with 10 μg/ml DEAE-Dextran were plated in 384 well clear-bottom plates (Greiner Bio-One GmbH, Germany). Luciferase reporter pseudovirus was spinoculated onto cells for 30min at 2095g and 4°C to settle virus onto cells but prevent receptor engagement. Following spinoculation the supernatant with un-adsorbed virus was removed and 70 μl of 37°C pre-warmed DMEM were added per well to initiate infection (time point zero) and plates were incubated at 37°C. At defined time points post-infection, 10 μl of the respective inhibitor were added to specific wells to stop the viral entry process. Inhibitors (T-20, Maraviroc, and DARPin 55.2) were added at saturating concentrations (final concentrations of 50 μg/ml, 5 μM and 1 μM, respectively) to ensure inhibition of entry. To obtain a measure for infectivity across different experiments, the wells with the last inhibitor addition at 120 minutes after infection start were used as 100% reference infectivity value and the infectivity of all other inhibitor treated wells were set in relation to it. In addition, a mock-treated well (addition of 10 μl DMEM at time point zero) was evaluated to assess absolute infectivity in absence of inhibitors. Luciferase reporter readout was performed after 48hr incubation at 37°C.

The time to reach 50% of the 120min infection (T½) was used as a surrogate for timing of receptor (CD4 or CCR5) binding or fusion. For each envelope, each inhibitor and each replicate, a general kinetic equation (A-D)/(1+/(x/C)^B) + D was fitted to each replicate time series of data points and a T½ value was estimated from the fitted equation. If the least-squares approximation used to fit the kinetic equation did not converge, a straight line was instead used to estimate T½. To deal with irregularities of the data, this line connects the data point left of the first point with >50% relative infectivity and the point right of the last point with <50% relative infectivity. The reported T½ value for each envelope and each inhibitor is the mean T½ value across all replicates (see Figs 6 and 7). Mann-Whitney tests were performed to compare the time intervals between the four stages of the entry process (synchronized start, CD4 binding, CCR5 attachment, fusion) among different envelopes. Only envelopes derived from the same patient were compared (see Fig 7 and S6 Fig).

To assess the sensitivity of the method used, we estimated T½ values in two different ways in addition to the method described above (see S7 Table). First, we took the mean or median of the data points from all replicate experiments prior to fitting only one curve and estimating T½. Since each experiment always consisted of two replicates, the second method took the mean or median of these two data points, fitted curves to these data, estimated T½ values for each pair and averaged them across experiments (see S7 Table). Whenever taking an average, we also considered using the median instead of the mean (see S7 Table).

### Estimating the stoichiometry of entry

The number of envelope glycoproteins required for target cell entry of the studied envelope variants was calculated as previously described [112–114]. In short, we generated dominant negative envelope genes in the pcDNA3.1 vector by knocking out the furin cleavage site (R508S/R511S). Ratios of the dominant negative mutant and wild-type envelopes were transfected into 293-T cells along with the NL-Luc-AM HIV-1 backbone to generate pseudotyped viral particles carrying varying ratios of functional and non-functional envelopes. Pseudovirus was then titrated on TZMbl target cells and read out after 48hrs using a Dynex MLX plate reader and Bright-Glo Luciferase Assay System (Promega, Madison Wisconsin, USA).

The number of envelope proteins per viral particle was estimated by quantitative ELISA of gp120 and p24, as previously described [112], and the mean trimer number of each virus variant determined. These data were then used to determine the number of trimers required for entry using the mathematical framework described in [112–114].

## Supporting information

S1 Table Inhibitors and antibodies. (TIF)

S2 Table Overview Env mutation in clones isolated from CD4^low^ and reversion culturing. (TIF)

S3 Table Multi-clade virus panel probed for CD4-DARPin 55.2 sensitivity. (TIF)

S4 Table CD4^low^ adaptation panel sequence characteristics. (TIF)

S5 Table Patient isolate pairs of macrophage- and non-macrophage-tropic envelopes. (TIF)

S6 Table Inhibitory activity of CD4 and CCR5 inhibitors against CD4^low^ adapted viruses during free-virus infection and fusion. (TIF)

S7 Table Entry kinetics curve fitting sensitivity analysis. (TIF)

S8 Table Estimations of trimer number per virion by quantitative gp120 and p24 ELISA ratios. (TIF)

S1 Fig Differential infection of Affinofile cells with varying CD4 and CCR5 levels by CD4^low^ adapted viruses. 293-T Affinofiles were induced to express forty-two unique combinations of (A) CD4 and (B) CCR5 levels. Receptor levels were assessed by quantitative flow cytometry. Data are from one of two independent experiments. (C) and (D) Infection of Affinofile matrices. Following receptor induction Affinofiles were infected with the indicated envelope pseudotyped viruses. For each pseudovirus, infection across the Affinofile matrix was normalized to the maximum infection this virus reached on Affinofiles in an individual experiment. Data from two independent experiments are shown, error bars = SD. (C) Mean percent maximum infections were plotted by CD4 level. Each panel indicates one level of CD4 induction with CCR5 levels increasing from left to right within each cluster of colored bars. (D) Mean percent maximum infections were plotted by CCR5 level. Each panel indicates one level of CCR5 induction with CD4 levels increasing from left to right within each cluster of colored bars.
(TIF)

S2 Fig Three dimensional Affinofile infection profiles. Affinofiles were induced to express forty-two unique combinations of CD4 and CCR5 and infected with the indicated Env-pseudoviruses. Data of the CD4^low^ envelope panel shown in S1 Fig and primary virus JR-FL for comparison are depicted. Two independent assays are shown. Axes legends are indicated at top right, dotted line projects into the page.
(TIF)

S3 Fig Comparison of free virus, cell-cell transmission and fusion capacity of CD4^low^ adapted viruses. Data correspond to Fig 3 and depict raw RLU values obtained for the depicted experiments (A) Titration of Env pseudoviruses on TZM-bl and PBMC. Infectivity of CD4^low^ adapted viruses in (B) cell-cell transmission and (C) fusion.
(TIF)

S4 Fig Infectivity of CD4^low^ adapted viruses on macrophages. Differentially conditioned monocyte derived macrophage phenotypes and infection. (A) Phenotypic verification of M-MDM and G-MDM preparation by phase contrast morphology and flow cytometry analysis of CD4, CD64 and CD163. Histograms depict one of 2 independent experiments for CD163 and CD64 staining, and one of 4 independent experiments for CD4 staining, dot-plot shows trends of CD4 and CCR5 staining levels for four independently isolated and treated batches of MDM. (B) Envelope pseudotyped virus stocks were freshly produced by transfecting 293-T cells with pcDNA3.1 envelope expression plasmid together with pNLluc-AM backbone and viral stocks titrated on M-MDM and G-MDM of eight different donors. Data show summary of experiments that were normalized either by input volume, RLU value determined by TZM-bl infectivity (RLU/μl), or p24 as determined by ELISA of viral stocks. Infection readout was normalized to NAB01. (C) Absolute infectivity of ultracentrifugation purified Env-pseudovirus stocks on differentially M-MDM and G-MDM with virus input normalized by p24 content. Mean, error bars = SD.
(TIF)

S5 Fig Prevalence of key mutations observed upon adaptation to CD4^low^ targets amongst primary viruses recorded in the Los Alamos sequence database. 4907 available Env sequences were analyzed.
(TIF)

S6 Fig Statistical analysis of entry kinetics following paired curve fitting. Alternative analysis of transition time between steps of the entry process of the data depicted in Fig 7. Data points from the two replicates from the same experiment were combined (i.e. paired) before fitting the curves and averaging individual T½ values. Estimated time intervals between the four stages of the entry process (synchronized start, CD4 binding, CCR5 attachment, fusion) were compared by Mann-Whitney tests. Only envelopes from the same patient (same principal color) were compared.
(TIF)

S7 Fig Comparison of free virus, cell-cell transmission and fusion capacity of patient-matched macrophage tropic and non-macrophage-tropic viruses. Data correspond to Fig 8 and depicts raw RLU values obtained for the (A) titration of Env pseudoviruses on TZM-bl and infectivity of CD4^low^ adapted viruses in (B) cell-cell transmission and (C) fusion.
(TIF)
