# Supplementary figures and images for: Delineating CD4 dependency of HIV-1: Adaptation to infect low level CD4 expressing target cells widens cellular tropism but severely impacts on envelope functionality
Source: PLoS Pathog. 2017 Mar 6;13(3):e1006255. doi: 10.1371/journal.ppat.1006255 (PMC5354460; doi:10.1371/journal.ppat.1006255)

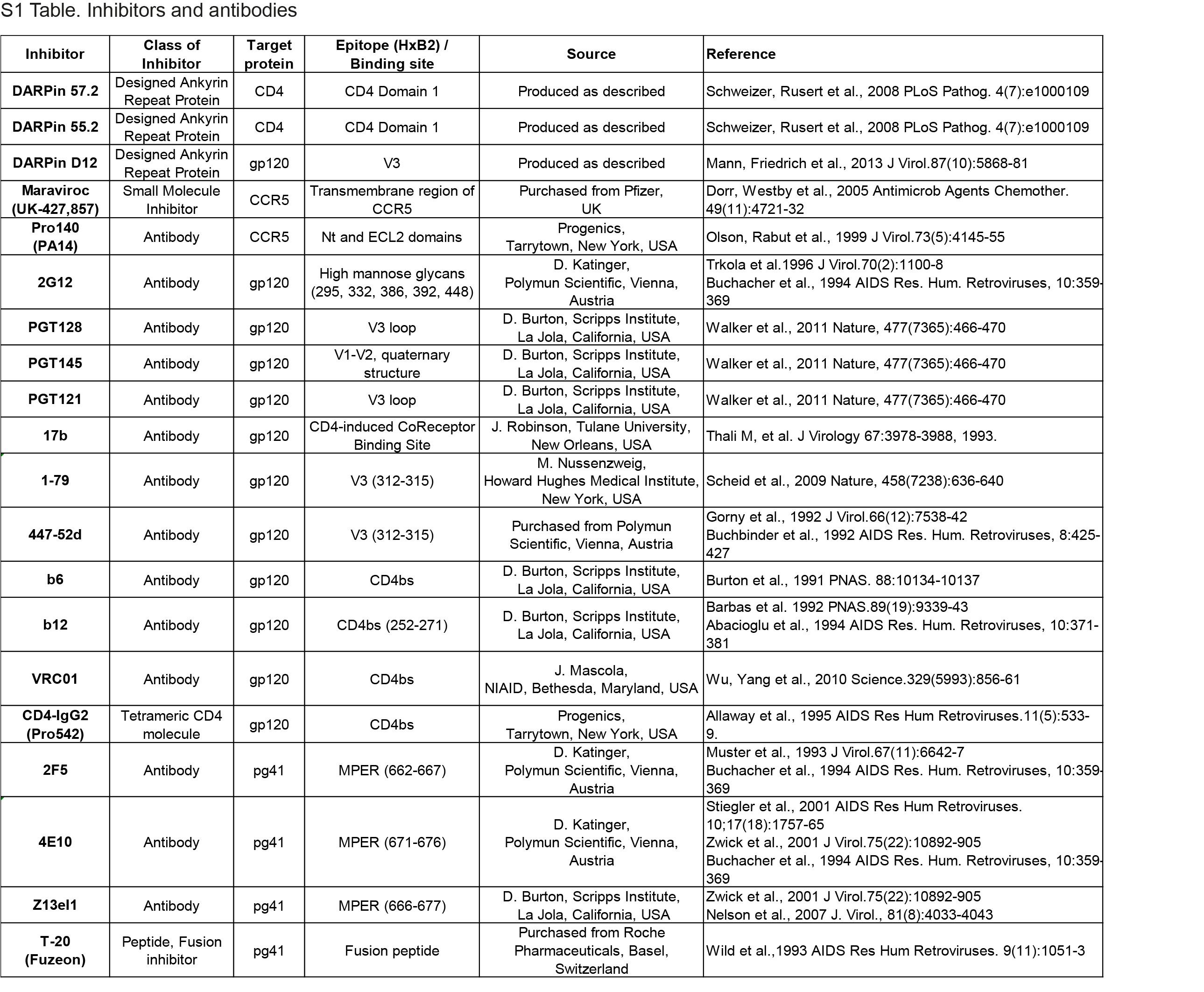

Supplement: S1 Table — (TIF) [file ppat.1006255.s001.tif]

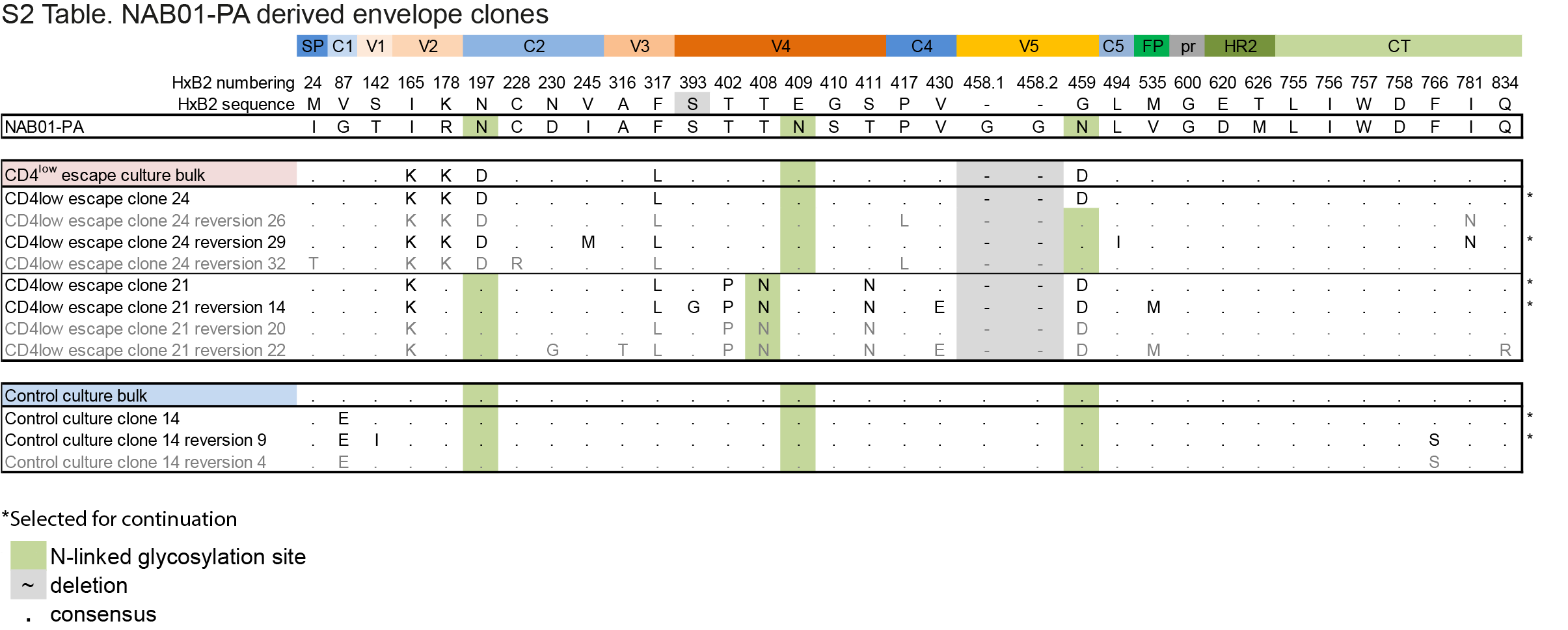

Supplement: S2 Table — (TIF) [file ppat.1006255.s002.tif]

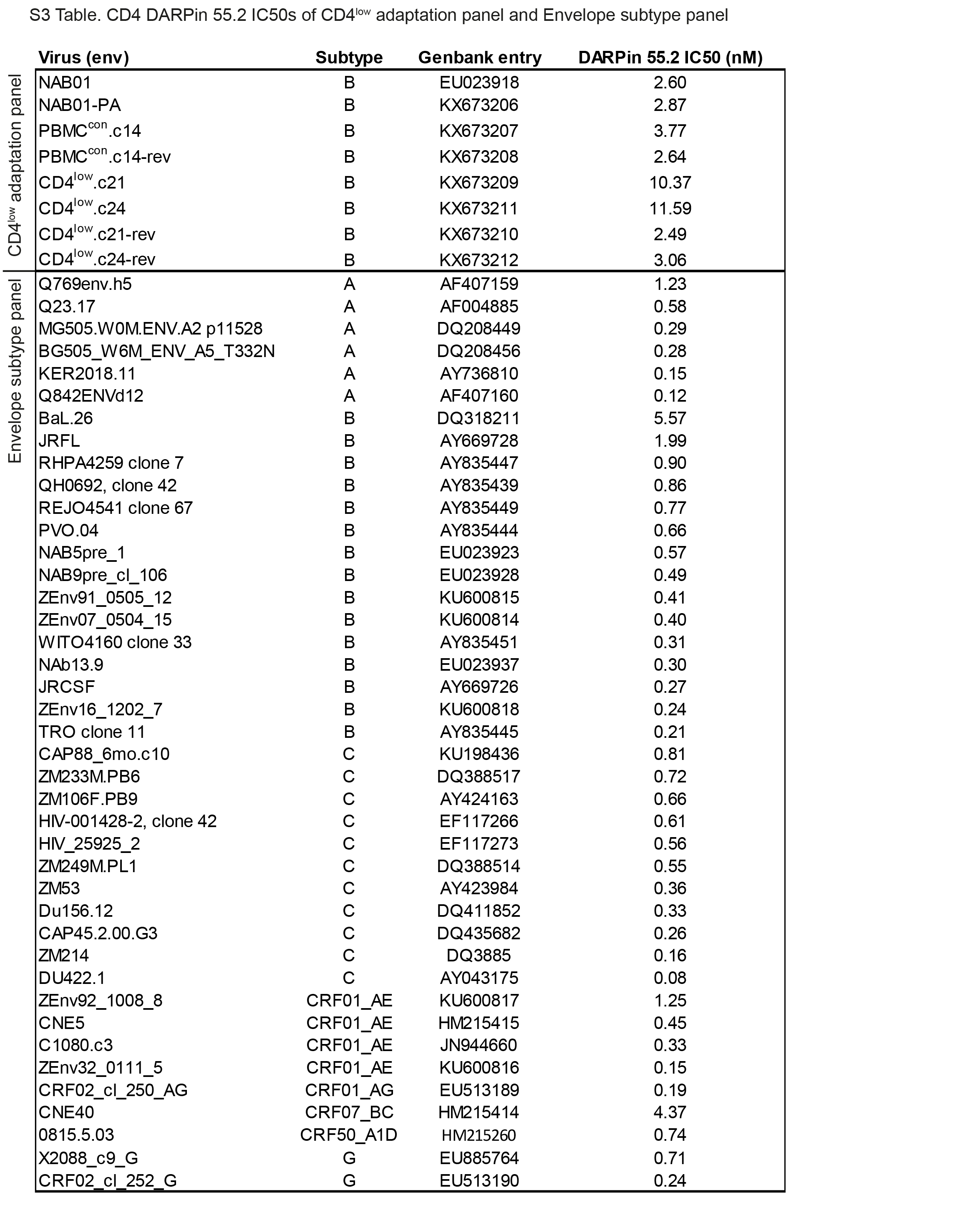

Supplement: S3 Table — (TIF) [file ppat.1006255.s003.tif]

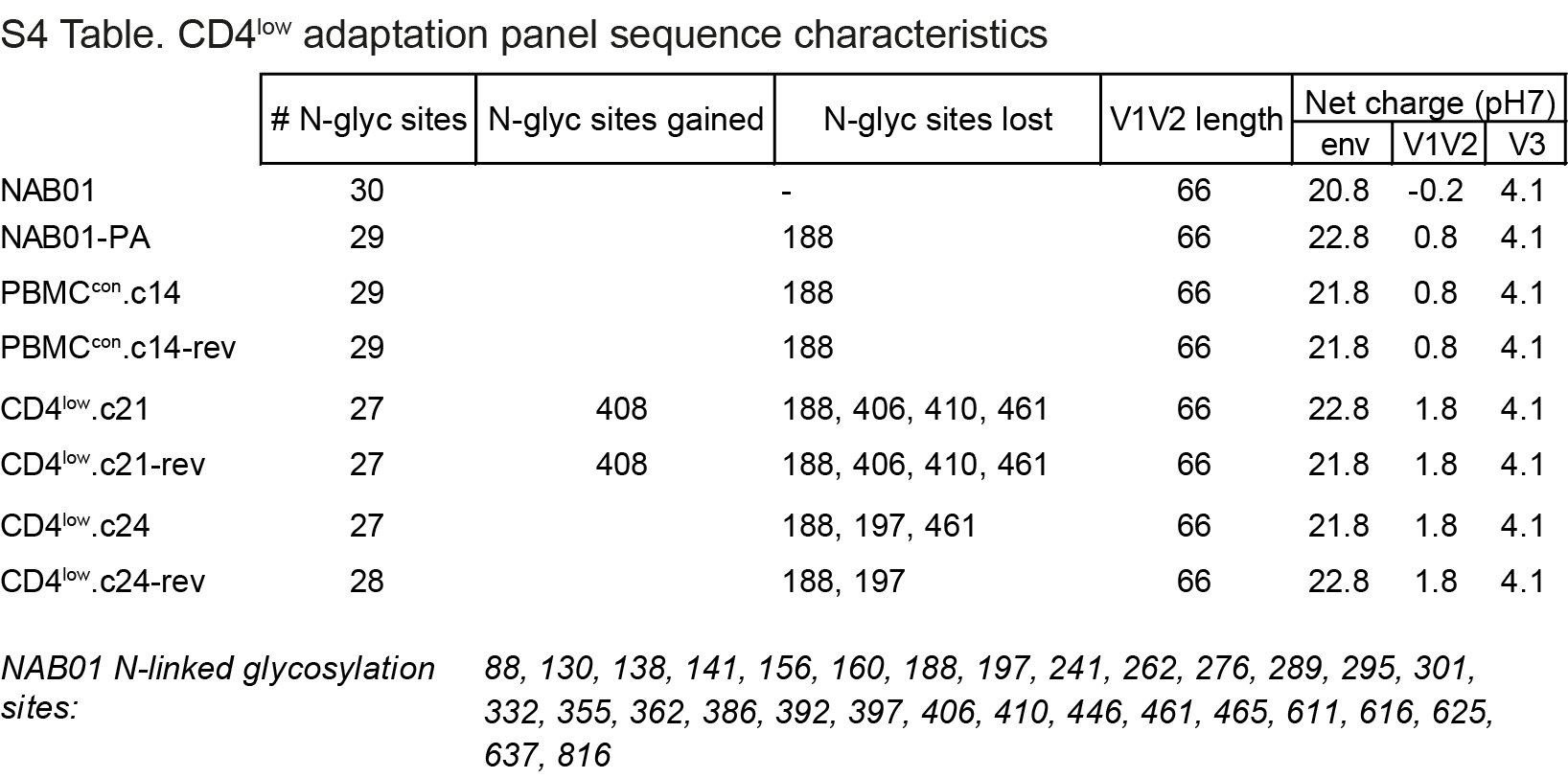

Supplement: S4 Table — (TIF) [file ppat.1006255.s004.tif]

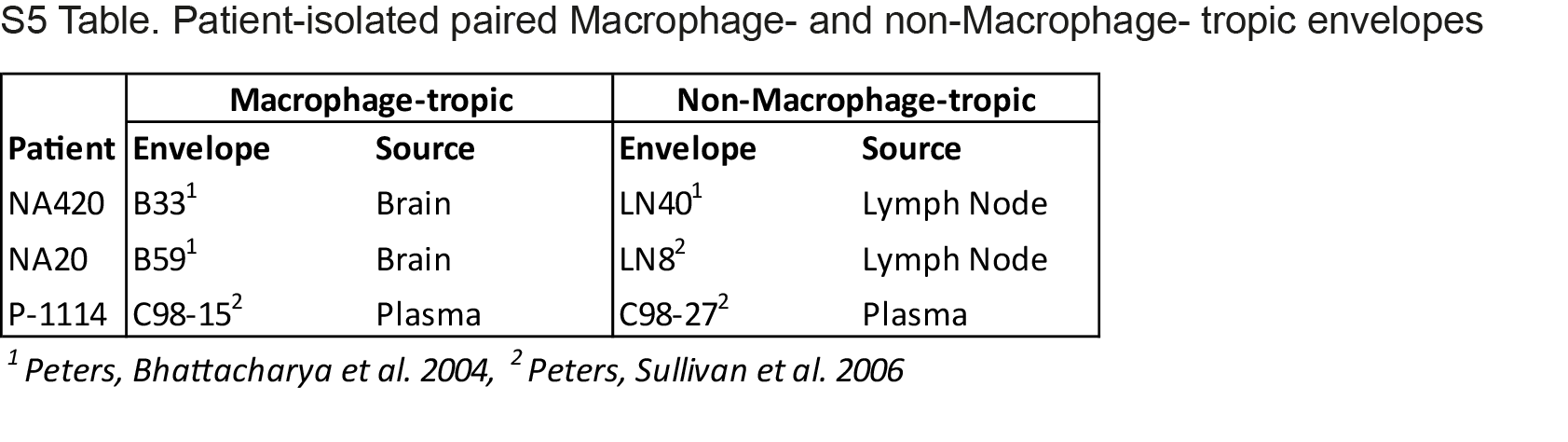

Supplement: S5 Table — (TIF) [file ppat.1006255.s005.tif]

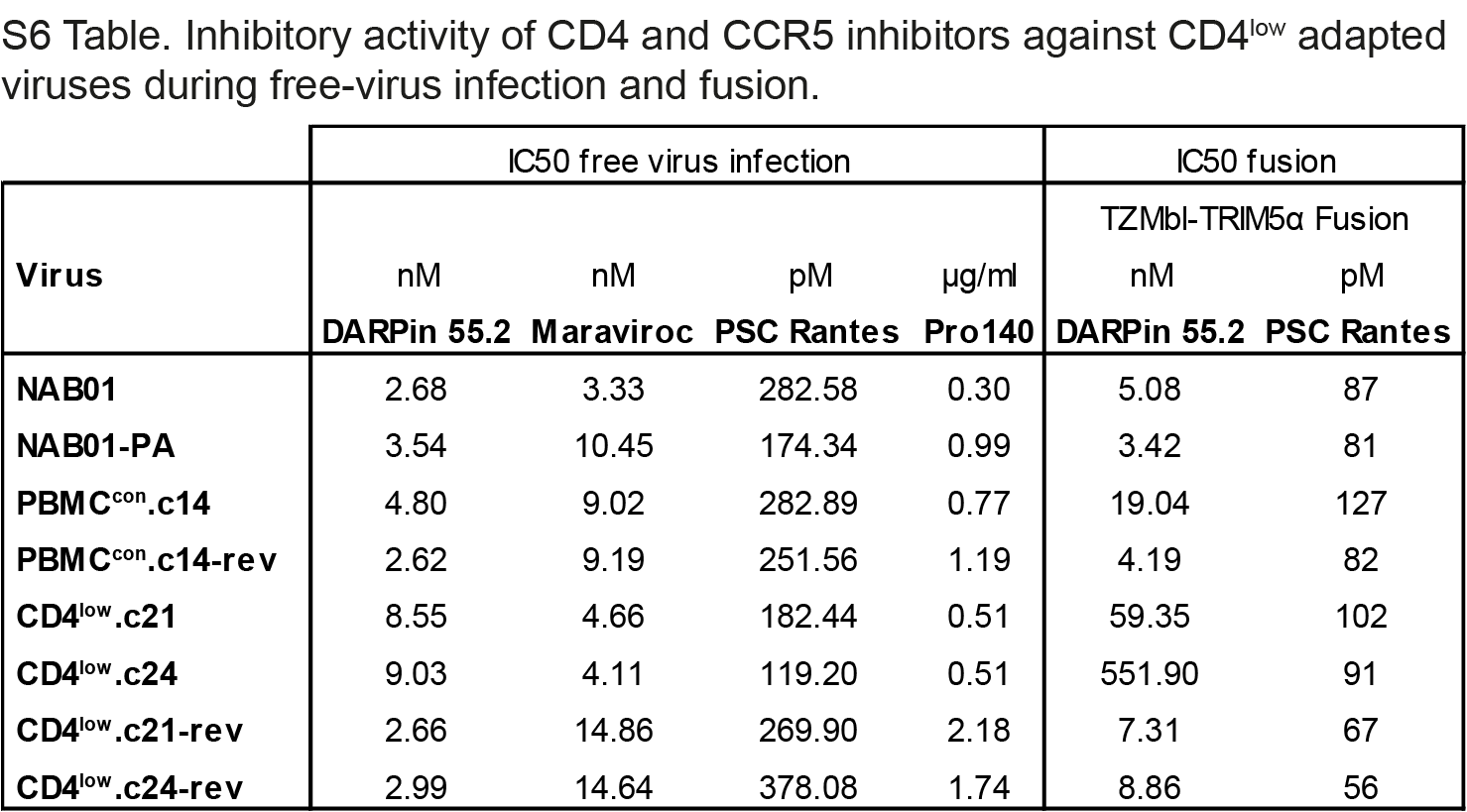

Supplement: S6 Table — (TIF) [file ppat.1006255.s006.tif]

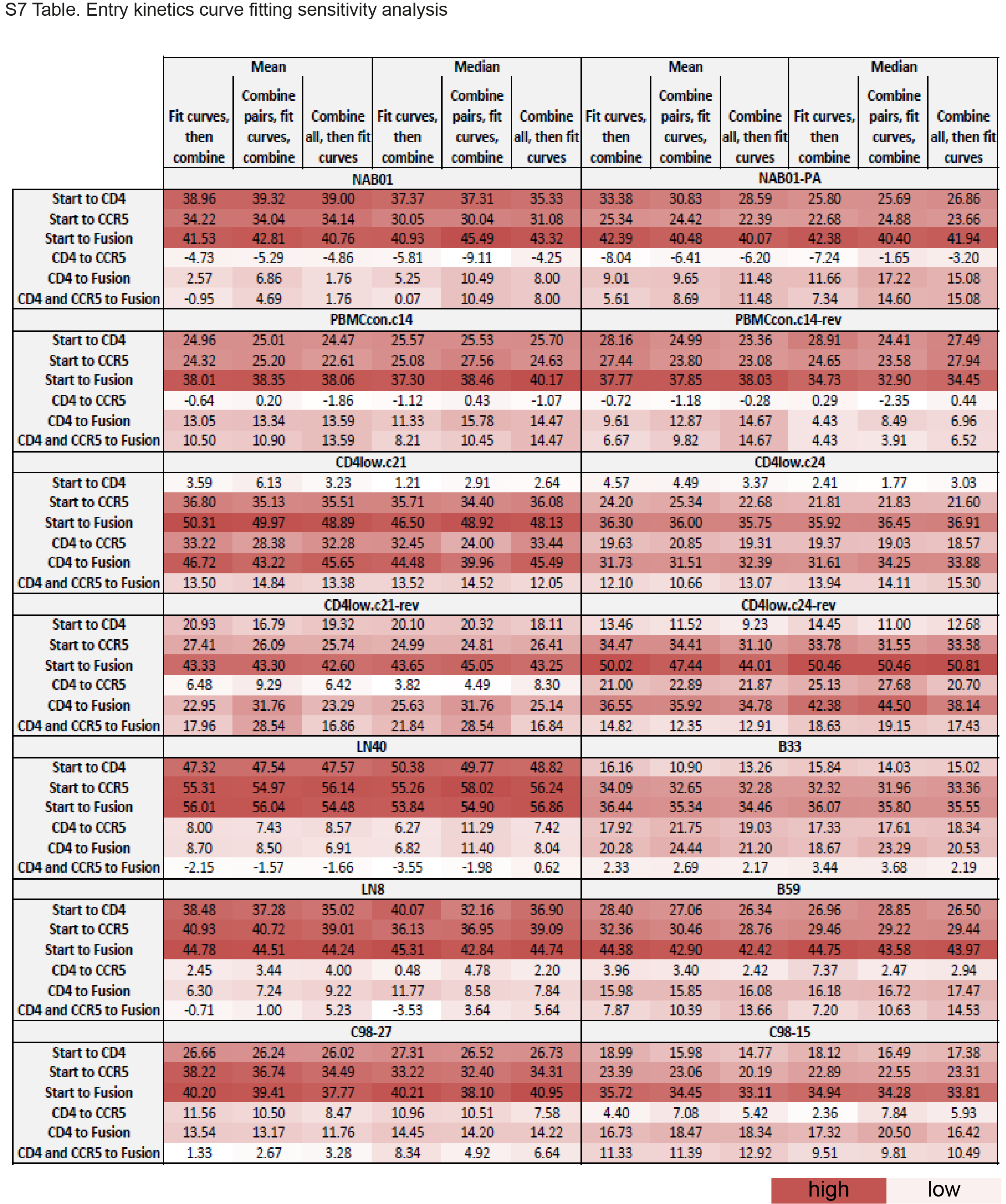

Supplement: S7 Table — (TIF) [file ppat.1006255.s007.tif]

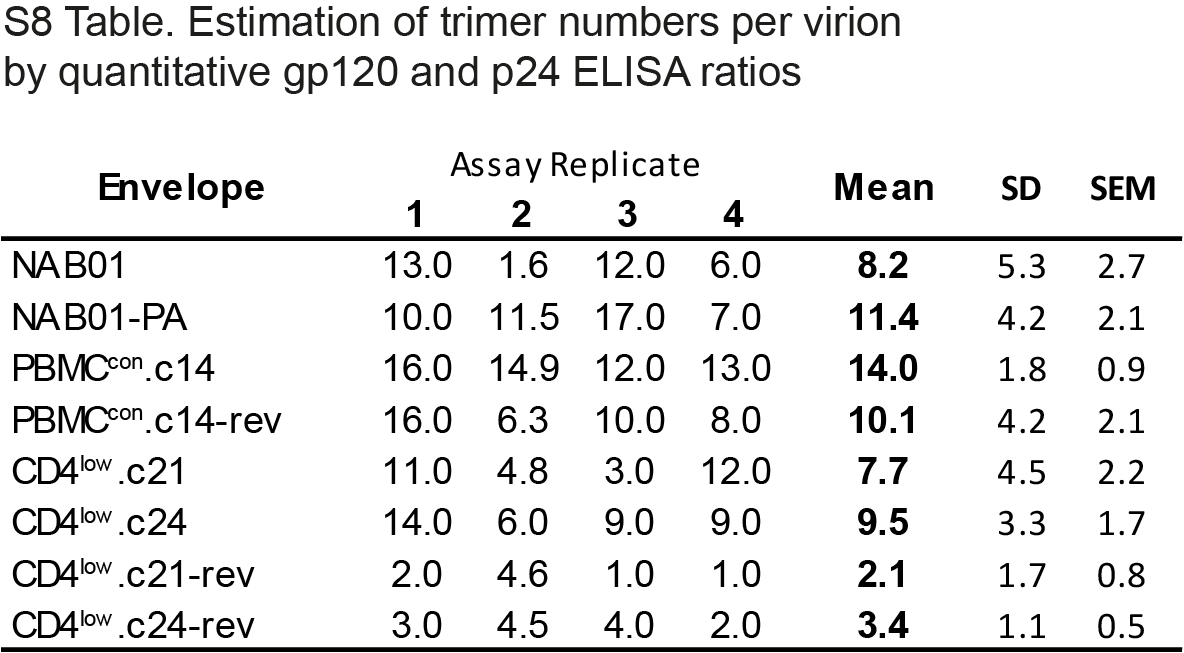

Supplement: S8 Table — (TIF) [file ppat.1006255.s008.tif]

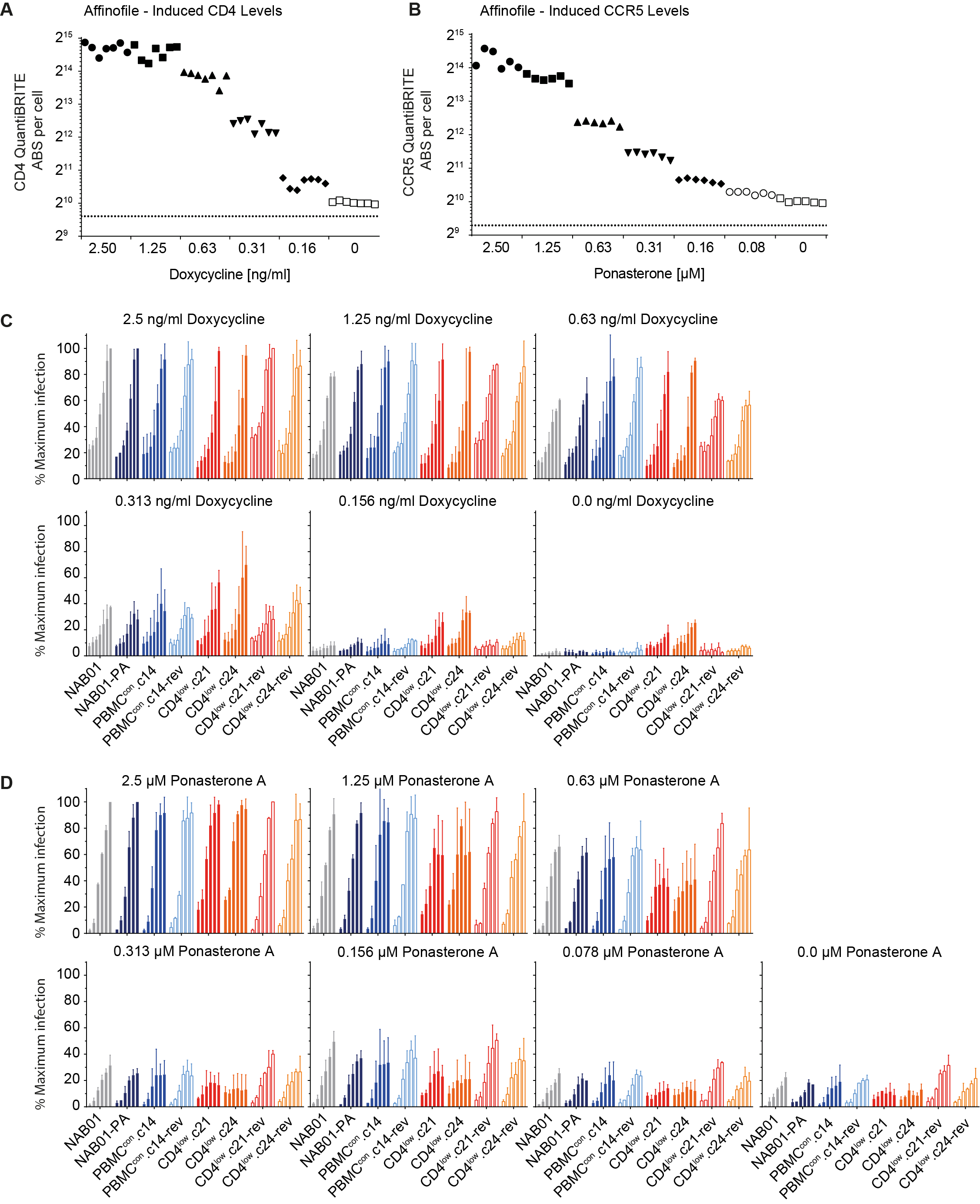

Supplement: S1 Fig — 293-T Affinofiles were induced to express forty-two unique combinations of (A) CD4 and (B) CCR5 levels. Receptor levels were assessed by quantitative flow cytometry. Data are from one of two independent experiments. (C) and (D) Infection of Affinofile matrices. Following receptor induction Affinofiles were infected with the indicated envelope pseudotyped viruses. For each pseudovirus, infection across the Affinofile matrix was normalized to the maximum infection this virus reached on Affinofiles in an individual experiment. Data from two independent experiments are shown, error bars = SD. (C) Mean percent maximum infections were plotted by CD4 level. Each panel indicates one level of CD4 induction with CCR5 levels increasing from left to right within each cluster of colored bars. (D) Mean percent maximum infections were plotted by CCR5 level. Each panel indicates one level of CCR5 induction with CD4 levels increasing from left to right within each cluster of colored bars. (TIF) [file ppat.1006255.s009.tif]

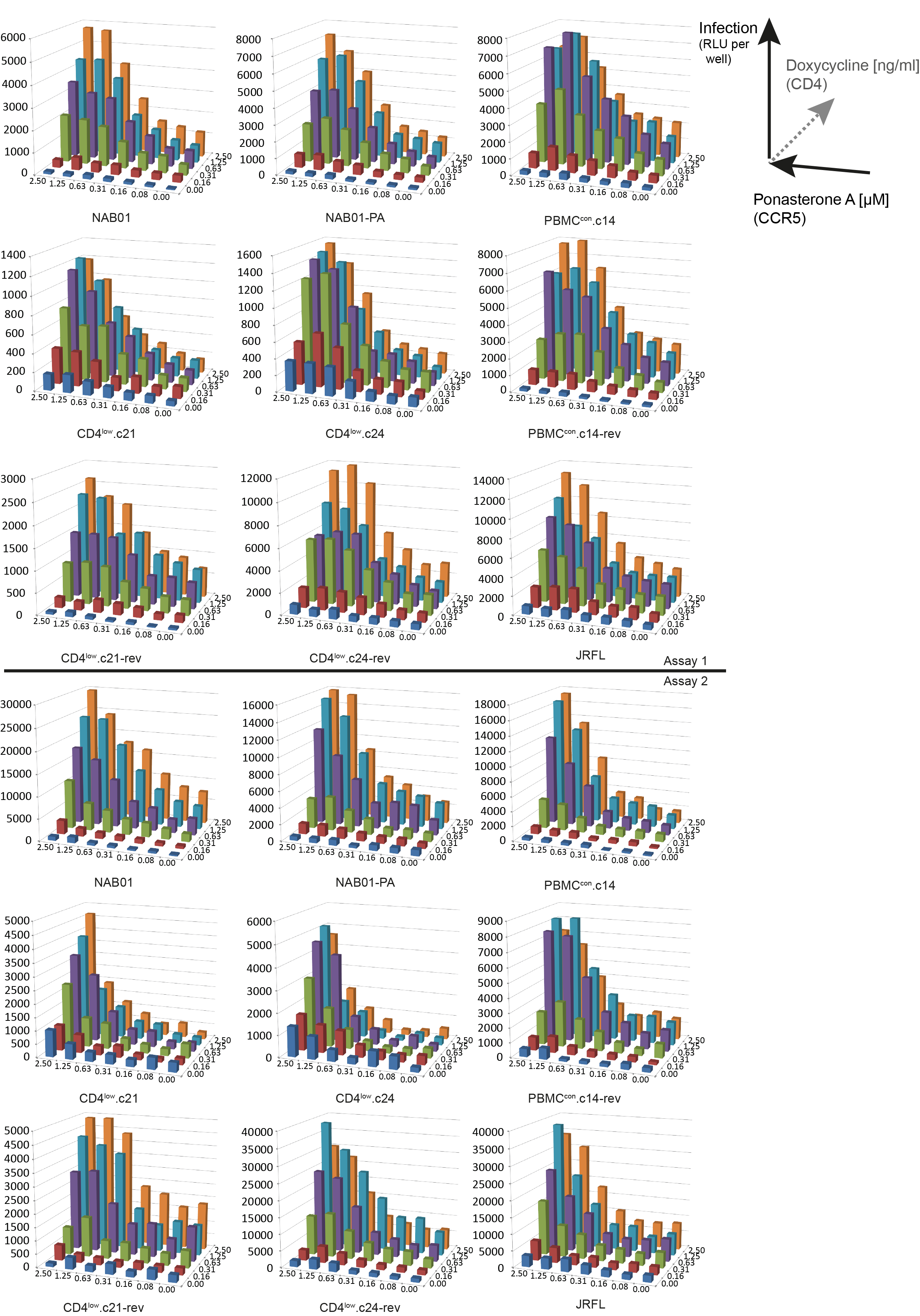

Supplement: S2 Fig — Affinofiles were induced to express forty-two unique combinations of CD4 and CCR5 and infected with the indicated Env-pseudoviruses. Data of the CD4low envelope panel shown in S1 Fig and primary virus JR-FL for comparison are depicted. Two independent assays are shown. Axes legends are indicated at top right, dotted line projects into the page. (TIF) [file ppat.1006255.s010.tif]

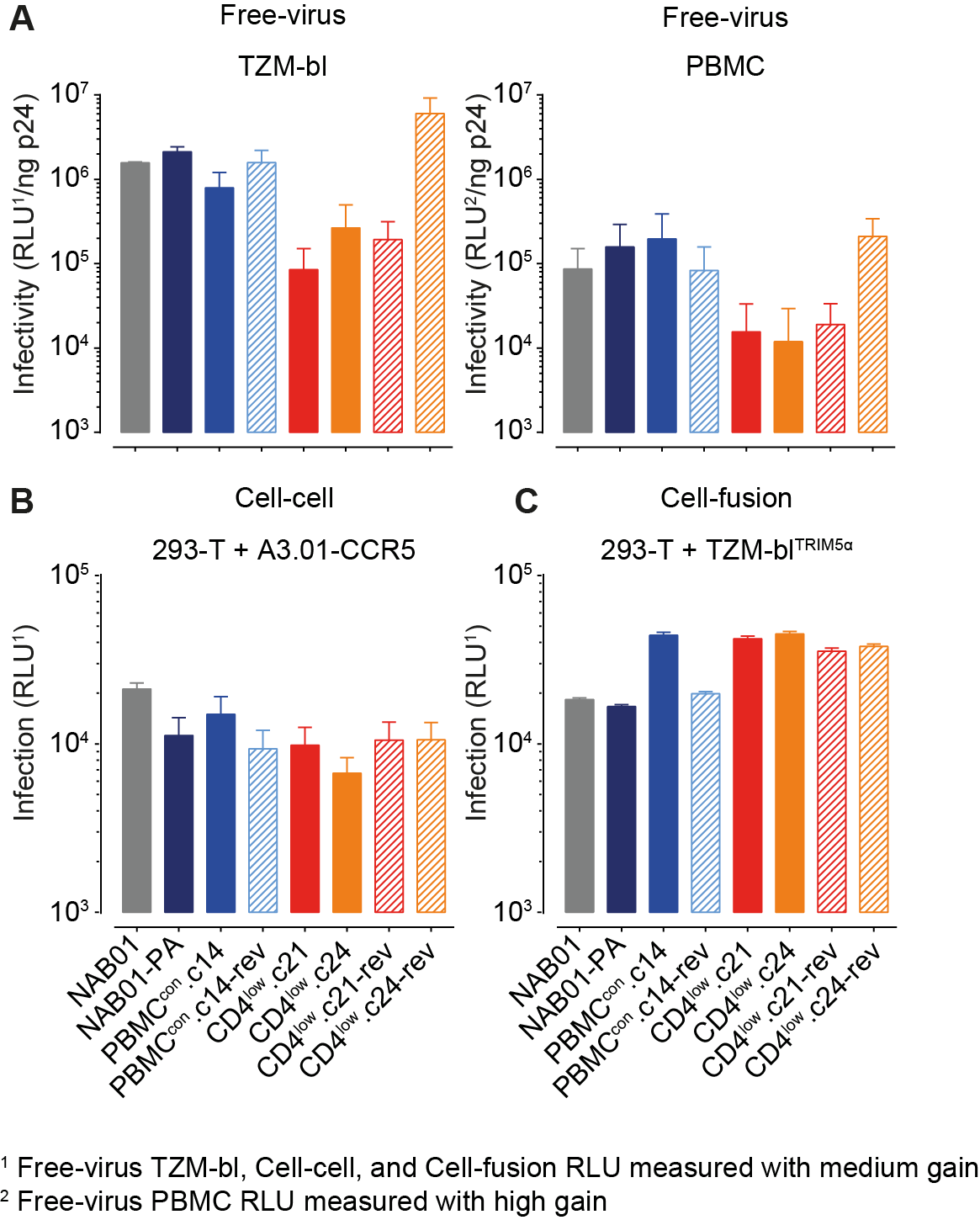

Supplement: S3 Fig — Data correspond to Fig 3 and depict raw RLU values obtained for the depicted experiments (A) Titration of Env pseudoviruses on TZM-bl and PBMC. Infectivity of CD4low adapted viruses in (B) cell-cell transmission and (C) fusion. (TIF) [file ppat.1006255.s011.tif]

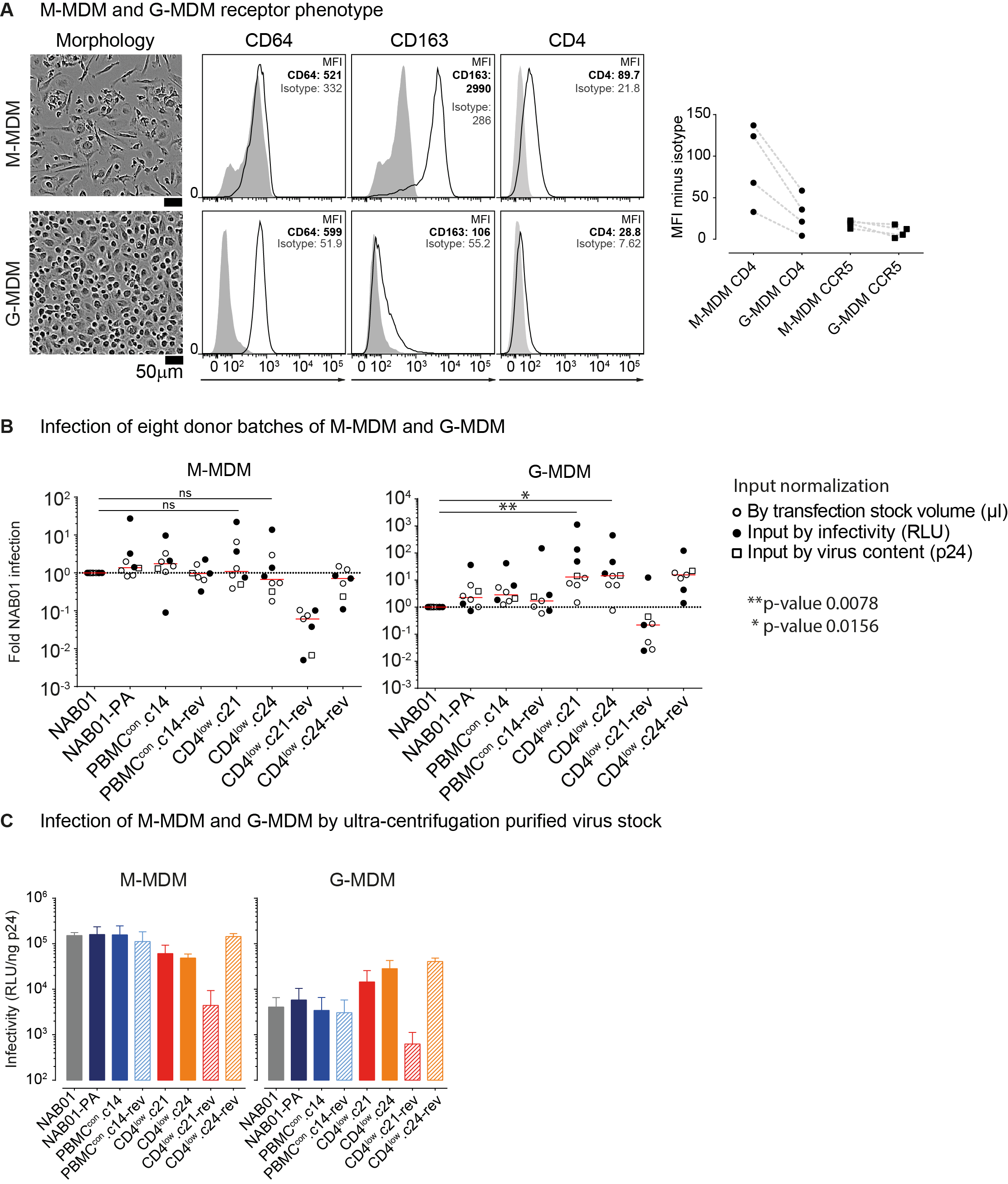

Supplement: S4 Fig — Differentially conditioned monocyte derived macrophage phenotypes and infection. (A) Phenotypic verification of M-MDM and G-MDM preparation by phase contrast morphology and flow cytometry analysis of CD4, CD64 and CD163. Histograms depict one of 2 independent experiments for CD163 and CD64 staining, and one of 4 independent experiments for CD4 staining, dot-plot shows trends of CD4 and CCR5 staining levels for four independently isolated and treated batches of MDM. (B) Envelope pseudotyped virus stocks were freshly produced by transfecting 293-T cells with pcDNA3.1 envelope expression plasmid together with pNLluc-AM backbone and viral stocks titrated on M-MDM and G-MDM of eight different donors. Data show summary of experiments that were normalized either by input volume, RLU value determined by TZM-bl infectivity (RLU/μl), or p24 as determined by ELISA of viral stocks. Infection readout was normalized to NAB01. (C) Absolute infectivity of ultracentrifugation purified Env-pseudovirus stocks on differentially M-MDM and G-MDM with virus input normalized by p24 content. Mean, error bars = SD. (TIF) [file ppat.1006255.s012.tif]

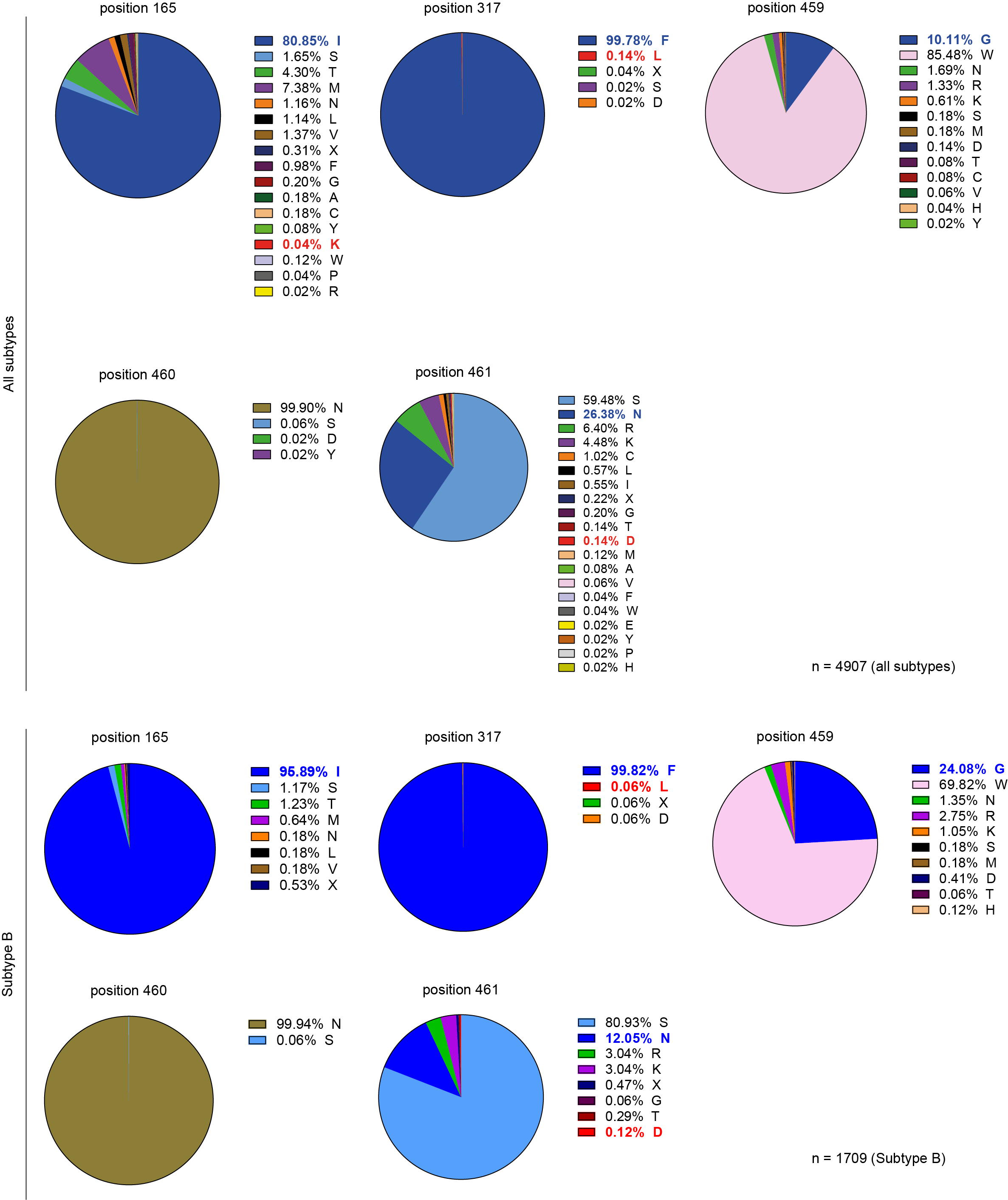

Supplement: S5 Fig — 4907 available Env sequences were analyzed. (TIF) [file ppat.1006255.s013.tif]

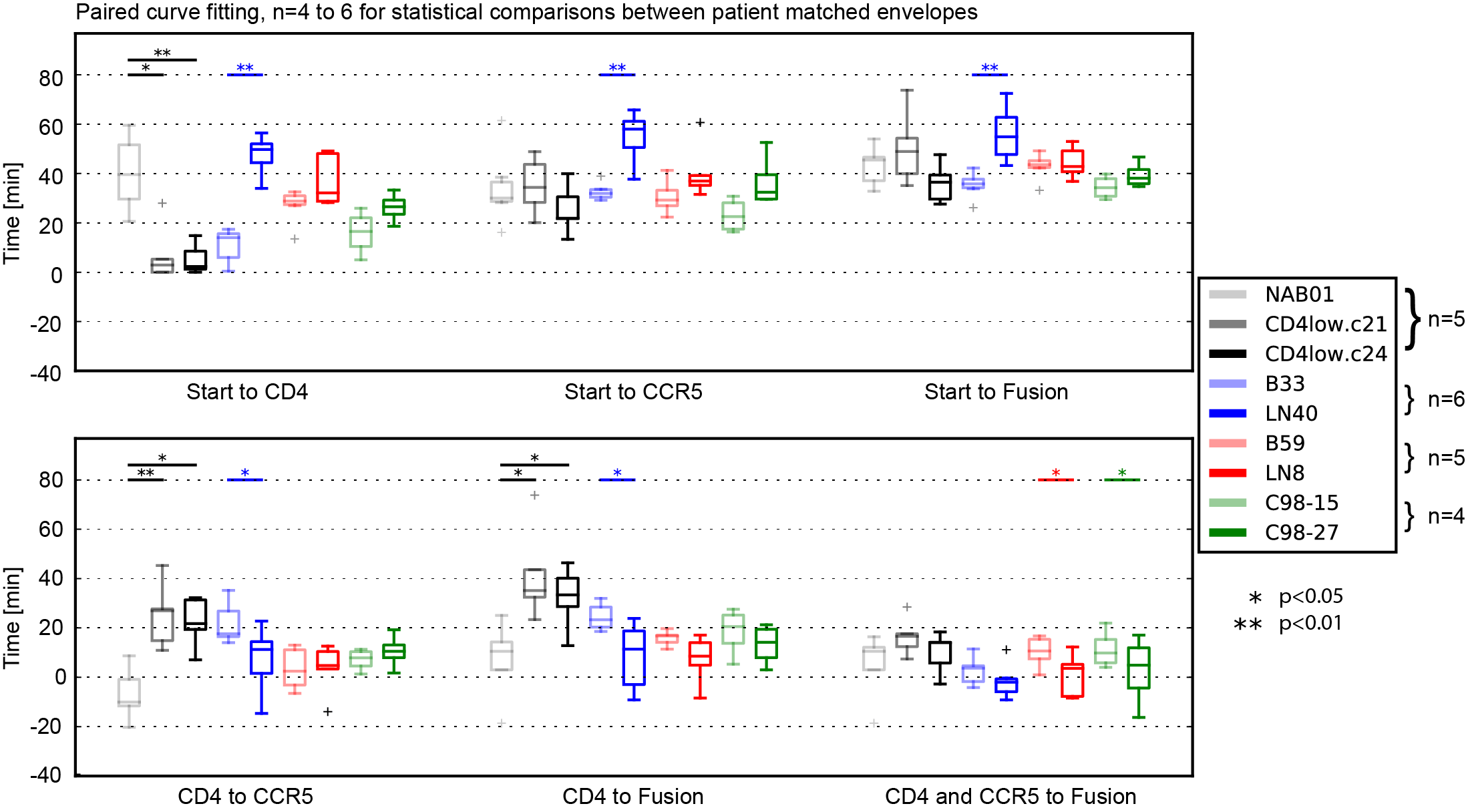

Supplement: S6 Fig — Alternative analysis of transition time between steps of the entry process of the data depicted in Fig 7. Data points from the two replicates from the same experiment were combined (i.e. paired) before fitting the curves and averaging individual T½ values. Estimated time intervals between the four stages of the entry process (synchronized start, CD4 binding, CCR5 attachment, fusion) were compared by Mann-Whitney tests. Only envelopes from the same patient (same principal color) were compared. (TIF) [file ppat.1006255.s014.tif]

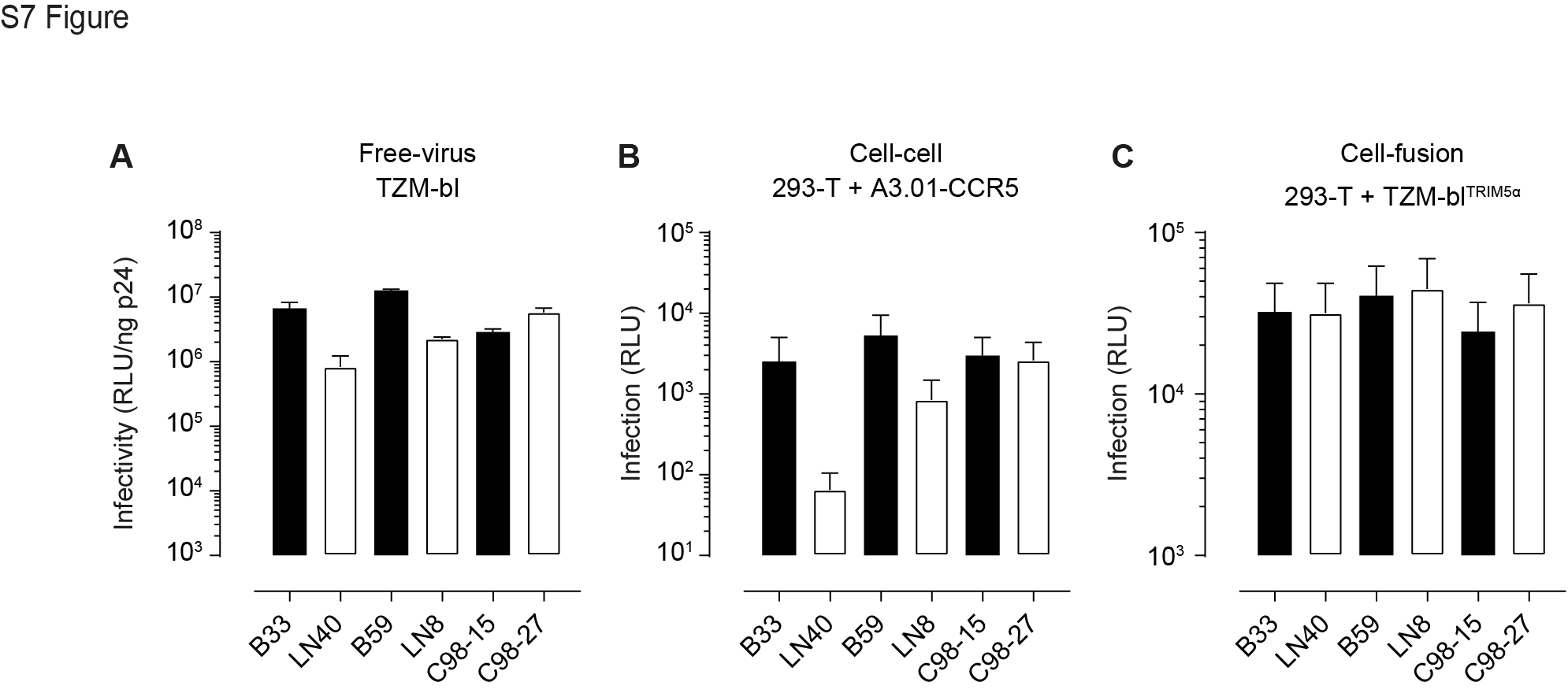

Supplement: S7 Fig — Data correspond to Fig 8 and depicts raw RLU values obtained for the (A) titration of Env pseudoviruses on TZM-bl and infectivity of CD4low adapted viruses in (B) cell-cell transmission and (C) fusion. (TIF) [file ppat.1006255.s015.tif]
